# Functional Imaging of Liver Cancer (FLIC): Study protocol of a phase 2 trial of ^18^F-DCFPyL PET/CT imaging for patients with hepatocellular carcinoma

**DOI:** 10.1371/journal.pone.0277407

**Published:** 2022-11-11

**Authors:** Esther Mena, Joanna Shih, Joon-Yong Chung, Jennifer Jones, Atoosa Rabiee, Cecilia Monge, Baris Turkbey, Liza Lindenberg, Kilian E. Salerno, Michael Kassin, Brad Wood, Jonathan Hernandez, Roberto Maass-Moreno, Babak Saboury, Neha Jakhete, Jason K. Molitoris, Keith R. Unger, Peter L. Choyke, Freddy E. Escorcia

**Affiliations:** 1 Molecular Imaging Branch, National Cancer Institute, NIH, Bethesda, MD, United States of America; 2 Biostatistics Branch, National Cancer Institute, NIH, Bethesda, MD, United States of America; 3 Radiation Oncology Branch, NCI, NIH, Bethesda, MD, United States of America; 4 Department of Hepatology, Veterans Affairs Medical Center, Washington, DC, United States of America; 5 Thoracic and GI Malignancy Branch, NCI, NIH, Bethesda, MD, United States of America; 6 Liver Cancer Program, NCI, NIH, Bethesda, MD, United States of America; 7 Interventional Oncology Branch, NCI, NIH, Bethesda, MD, United States of America; 8 Surgical Oncology Branch, NCI, NIH, Bethesda, MD, United States of America; 9 Department of Radiology and Imaging Sciences, NCI, NIH, Bethesda, MD, United States of America; 10 Veterans Affairs Medical Center, Baltimore, MD, United States of America; 11 Department of Radiation Oncology, University of Maryland Medical Center, Baltimore, MD, United States of America; 12 Department of Radiation Medicine, MedStar Georgetown University Hospital, Washington, DC, United States of America; GERMANY

## Abstract

**Background:**

While prostate specific membrane antigen (PSMA) is overexpressed in high-grade prostate cancers, it is also expressed in tumor neovasculature and other malignancies, including hepatocellular carcinoma (HCC). Importantly, no functional imaging for HCC is clinically available, making diagnosis and surveillance following local therapies particularly challenging. ^18^F-DCFPyL binds with high affinity to PSMA yet clears rapidly from the blood pool. PET imaging with ^18^F-DCFPyL may represent a new tool for staging, surveillance and assessment of treatment response in HCC. The purpose of this Functional Imaging Liver Cancer (FLIC) trial is to assess the ability of ^18^F-DCFPyL-PET/CT to detect sites of HCC.

**Methods:**

This is a phase II multi-site prospective imaging trial with a plan to enroll 50 subjects with suspected HCC on standard of care CT or MRI and eligible for standard local treatment. Participants will undergo a baseline ^18^F-DCFPyL-PET/CT, prior to therapy. Subjects will also be scanned with ^18^F-FDG-PET/CT within 2 weeks of ^18^F-DCFPyL-PET/CT. Participants will undergo histopathologic assessment and standard of care local treatment for HCC within a multidisciplinary team context. Participants with histopathologic confirmation of HCC and a positive baseline ^18^F-DCFPyL-PET/CT will undergo a post-treatment ^18^F-DCFPyL-PET/CT during the first routine follow-up, typically within 4–8 weeks. Subjects with negative baseline ^18^F-DCFPyL-PET/CT will not be re-scanned after treatment but will remain in follow-up. Participants will be followed for 5-years to assess for progression-free-survival. The primary endpoint is the positive predictive value of ^18^F-DCFPyL-PET for HCC as confirmed by histopathology. Secondary endpoints include comparison of ^18^F-DCFPyL-PET/CT with CT, MRI, and ^18^F-FDG-PET/CT, and evaluation of the value of ^18^F-DCFPyL-PET/CT in assessing treatment response following local treatment. Exploratory endpoints include next generation sequencing of tumors, and analysis of extracellular vesicles to identify biomarkers associated with response to therapy.

**Discussion:**

This is a prospective imaging trial designed to evaluate whether PSMA-PET/CT imaging with ^18^F-DCFPyL can detect tumor sites, assess local treatment response in HCC patients, and to eventually determine whether PSMA-PET/CT could improve outcomes of patients with HCC receiving standard of care local therapy. Importantly, this trial may help determine whether PSMA-selective radiopharmaceutical therapies may be beneficial for patients with HCC.

**Clinical trial registration:**

NIH IND#133631. Submission date: 04-07-2021. Safe-to-proceed letter issued by FDA: 05.07.2021. NIH IRB #00080. ClinicalTrials.gov Identifier NCT05009979. Date of Registry: 08-18-2021. Protocol version date: 01-07-2022.

## Introduction

### Background

#### PSMA in hepatocellular carcinoma

Prostate specific membrane antigen (PSMA), also known as folate hydrolase 1 (FOLH1) or glutamate carboxypeptidase II, is a type II transmembrane glycoprotein extensively studied in prostate cancers. PSMA is overexpressed in high-grade prostate tumors, and increases when de-differentiation, metastatic or hormone-refractory disease occur, making PSMA prognostically significant for this disease [1–3]. However, PSMA is not entirely prostate-specific, and it is known to be expressed in normal tissues such as the lacrimal and salivary glands, in several other neoplasms, as well as in tumor-associated neovasculature [4, 5].

Hepatocellular carcinoma (HCC) accounts for about 80% of all primary liver cancers [6]. It is the fifth most common cancer world-wide and the third most common cause of cancer-related mortality [7]. Current guidelines of the American Association for the Study of Liver Disease and the European Association for the Study of the Liver recommend HCC surveillance with abdominal ultrasound every 6 months in participants at high risk of developing the disease. Patients diagnosed in early stages of HCC are eligible for potentially curative therapy; therefore, early diagnosis and accurate staging are critical for patient outcomes. Curative-intent therapies for HCC include surgical resection, liver transplantation, microwave ablation (MWA) and radiofrequency ablation (RFA), respectively—in case of small tumors [8]. Recent level 1 data for ablative stereotactic body radiotherapy (SBRT) demonstrated non-inferiority compared to radiofrequency ablation for small HCC lesions, suggesting an emerging curative role for this modality [9].

Imaging of HCC at diagnosis is challenging and exacerbated after local treatment because it can be confounded by coagulative necrosis, hematomas, abscesses, and fluid or bile collections [10]. Standard of care imaging modalities used routinely in the diagnosis of HCC are ultrasound (with or without contrast), multiphasic computed tomography (CT) or magnetic resonance imaging (MRI). Each method has its shortcomings, the major one being the lack of correlation between radiologic appearance and biologic activity. Furthermore, imaging of liver lesions in patients with concomitant liver cirrhosis is complex and challenging. The cirrhotic liver acquires a nodular architecture with altered vascularity, making it difficult to differentiate regenerative nodules from early HCC or metastases from other primary tumors. The challenge is exacerbated in patients who undergo ablative (RFA, MWA, SBRT) or transarterial radio-, chemo-, or bland embolization (TARE, TACE, TAE) therapies for known HCC lesions.

Hence, there is a need for an imaging modality that can be used to localize and characterize HCC tumors more accurately. To date, functional imaging with Positron Emission Tomography (PET) has not played a major role in the diagnosis or surveillance of HCC. The most commonly used PET tracer, F-18 Fluoro-deoxy-glucose (^18^F-FDG), is not routinely used in HCC because only a minority of tumors show FDG avidity [11] and its sensitivity (< 50%) for detection of HCC tumors is inferior to CT alone [12]. Other PET tracers have been tested in HCC, however, results have been mixed and none have been routinely used clinically [13].

#### Value of PSMA PET/CT in staging and therapy monitoring of HCC

Several PET radiotracers, including small molecule inhibitors targeting the PSMA receptor have been developed to facilitate the diagnosis of prostate cancer in humans [14–16]. PSMA PET/CT imaging with ^68^Ga-PSMA-11 and ^18^F-DCFPyL are such tracers approved for imaging patients with prostate cancer [17, 18]. Notably, PSMA expression has been demonstrated on other malignant cell lines and tumor-associated neovasculature. According to two reports, more than 90% of hepatocellular cancers stain positive for PSMA in the tumor vasculature [19, 20]. Zhu et al. suggested that the process of endothelial cell recruitment to HCC occurs early and throughout the process of hepatic tumorigenesis, making an endothelial cell tracer an ideal marker for early disease detection [21]. In another study assessing 103 HCC tissue samples using immunohistochemical staining, Jiao and collaborators reported PSMA expression in more than 74% of tumor vasculature [22]. Authors found that high vascular PSMA expression was associated with poor prognosis in patients with HCC, which could serve as an independent prognostic marker for HCC [22]. Tolkach et al. found that the majority of hepatocellular carcinomas show high levels of PSMA expression on the tumor neovasculature (89.9% of tumors) and on canalicular membrane of the tumor cells (41.1% of the tumors) [23].

To date, most publications involving PSMA-targeted PET radiotracers in HCC have been of case reports and small cohort studies [24, 25]. In a small cohort study (n = 7), Kesler et al. showed superiority of ^68^Ga-PSMA PET over ^18^F-FDG PET-CT in imaging newly diagnosed HCC [25]; all tumors, except for one lesion showed abnormal ^68^Ga-PSMA uptake higher than that of the surrounding liver parenchyma, with a mean uptake 3.6 times higher than the normal surrounding liver background. The increased ^68^Ga-PSMA uptake in HCC was corroborated by the results of immunohistochemistry analysis showing PSMA staining of the endothelial cell lining of vessels that are penetrated by tumor. Furthermore, ^68^Ga-PSMA PET appeared to differentiate necrotic from viable tumor, which could dramatically improve detection of HCC recurrence following local ablative therapies [25]. In another study, Kuyumcu et al. were able to visualize advanced HCC using ^68^Ga-PSMA-PET/CT in 16 out of 19 cases with high tumor-to-background ratio [26]. Hirmas et al. compared ^68^Ga-PSMA-11 PET and CT accuracy for HCC lesion detection and assessed the impact of PSMA PET on management and prognosis in 40 patients. Investigations found comparable accuracy between ^68^Ga-PSMA-11 PET and CT for staging at the liver level, with superior performance for ^68^Ga-PSMA-11 PET at the extrahepatic level. PSMA PET/CT accuracy was associated with change in management, particularly in patients with advanced disease, leading to a shift toward systemic therapy [27]. A recent prospective study on 15 patients with HCC reported improved lesion detection with ^68^Ga-PSMA-11 PET/CT compared with conventional imaging and a subsequent impact on treatment strategies [28]. The largest prospective study to date was performed by Thompson et al., which first assessed PSMA staining of both cholangiocarcinoma and HCC specimens and PET imaging with ^68^Ga-PSMA-11 [20]. This study showed that 91% of HCC exhibited positive PSMA staining, and 64% of suspected HCC lesions exhibited ^68^Ga-PSMA-11 tracer uptake (39 lesions in 31 patients). Because only 7 patients underwent both pathologic confirmation and PSMA imaging, the discrepant results may reflect differences of PSMA expression, non-HCC diagnoses, or both.

Few direct comparisons between ^18^F-DCFPyL and ^68^Ga-PSMA-PET have been reported in prostate cancer populations. Dietlein et al. reported comparable biodistributions for both tracers, but slightly higher sensitivity for ^18^F-DCFPyL compared to ^68^Ga-PSMA-11 (88% vs 66%) in patients with biochemically recurrent prostate cancer [29].

Although we live in the era of precision oncology, currently, there are no tumor- or tumor microenvironment-targeted therapeutic agents for HCC. ^18^F-DCFPyL could be the functional imaging agent urgently needed in the clinic and it is being evaluated in our Functional Imaging of Liver Cancer (FLIC) National Cancer Institute study (NCT05009979), at the Mayo Clinic, in Rochester, Minnesota (NCT04310540 and NCT04762888), at the Peter MacCallum Cancer Centre in Melbourne, Australia (NCT05095519) and at Wuhan Union Hospital, China (NCT05006326).

Notably, if PSMA PET imaging, indeed, serves as useful for HCC, we could deploy therapeutic PSMA-specific ligands either intravenously, or perhaps trans-arterially, in line with the “theranostics” approach, which employs molecular imaging to guide molecularly directed radiopharmaceutical therapy.

### Rationale for study design and hypothesis

**Fig 1:**
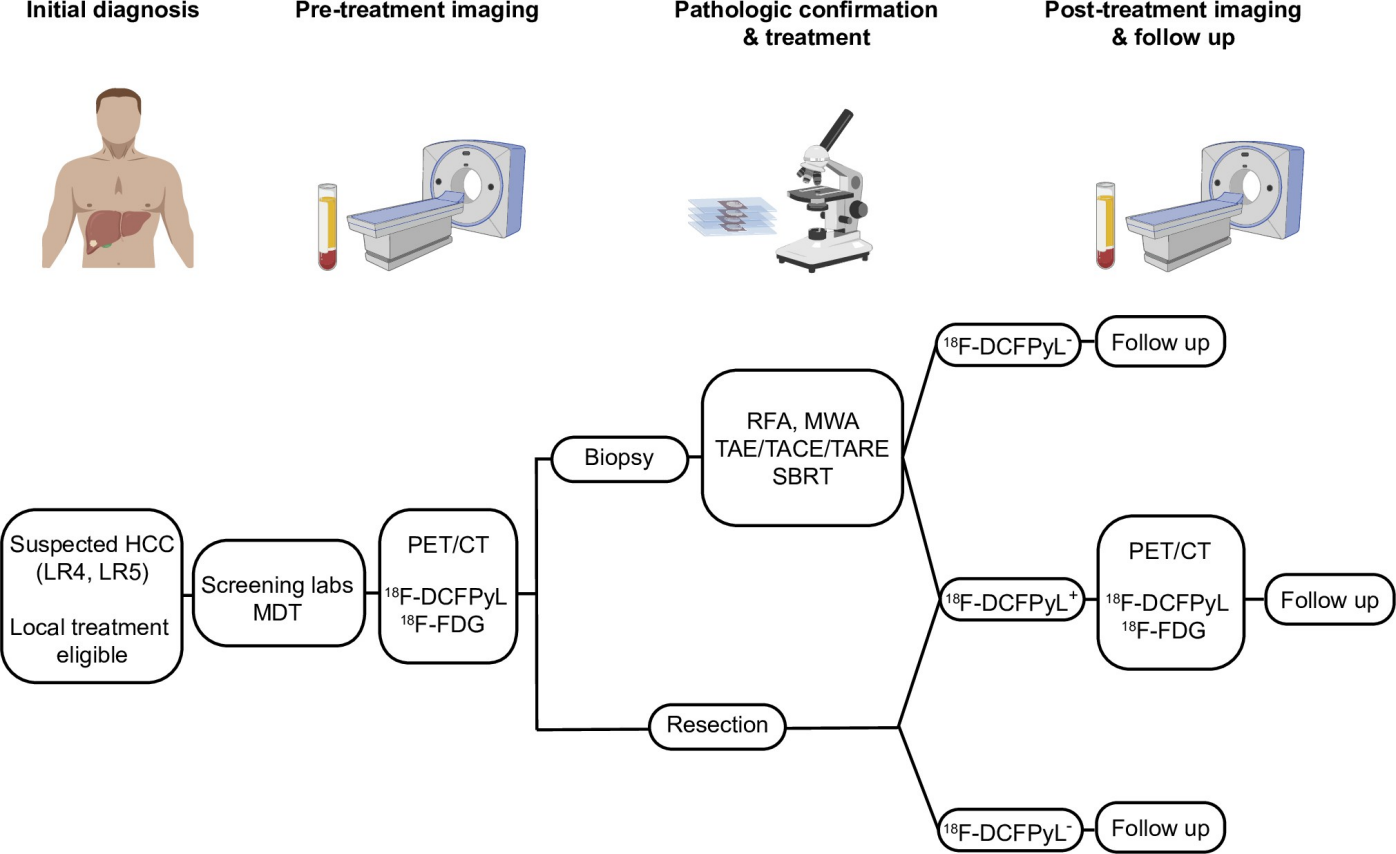
Protocol schema. Patients who have LIRADS 4 or 5 lesions on standard imaging (CT or MRI) and deemed good candidates for any local treatment are eligible for this study. Prior to treatment, PET/CT with both ^18^F-DCFPyL and ^18^F-FDG will be performed. Histopathologic confirmation of HCC will be performed on either biopsy or surgical specimen. Only patients who had positive ^18^F-DCFPyL PET/CT pre-treatment will undergo post-treatment ^18^F-DCFPyL PET/CT (2–3 months post treatment) to determine its utility as a functional imaging marker. Scale bar shown is 100 *μ*m. The images were created with BioRender.com, with permission privileges.

**Fig 2:**
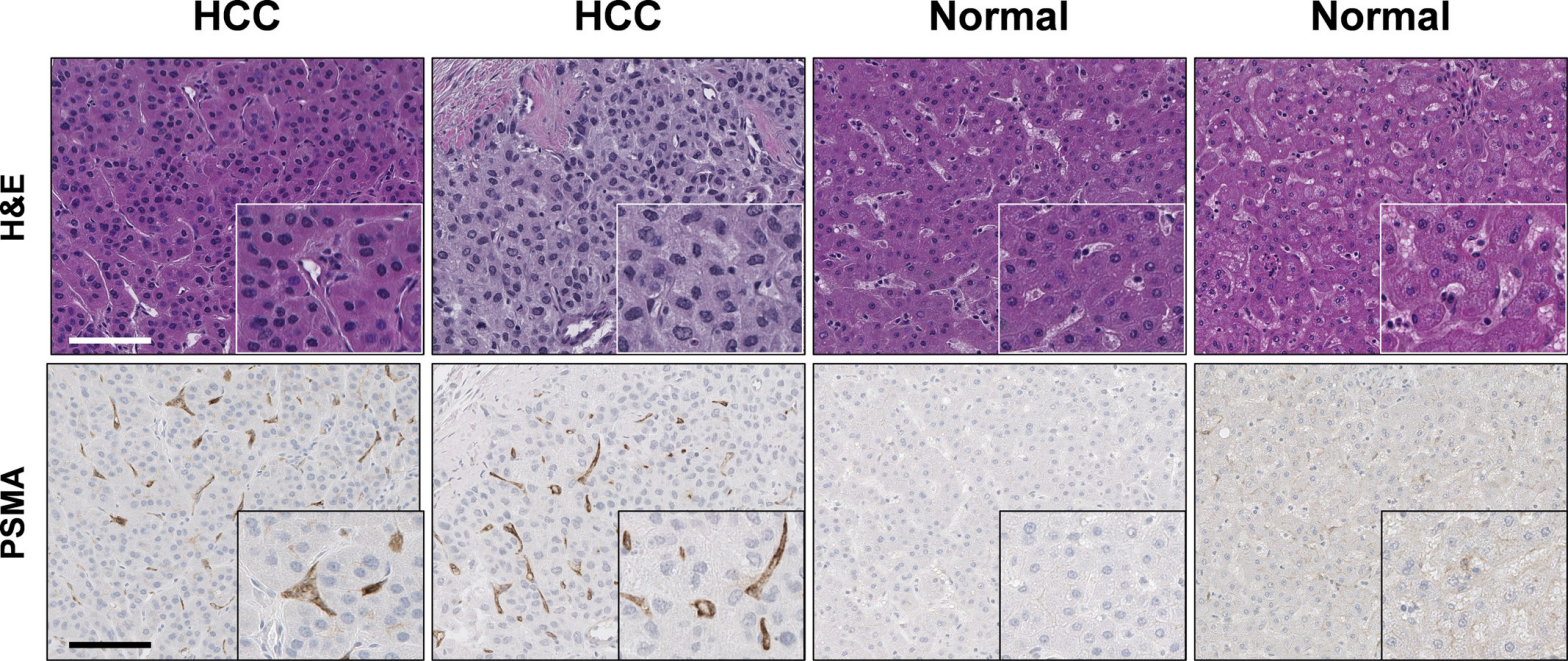
Representative immunohistochemical staining for prostate-specific membrane antigen (PSMA) in formalin-fixed paraffin-embedded liver tissues. PSMA expression was assessed using anti-PSMA antigen antibody (Cell Signaling, Danvers, MA; D718E clone) and avidin-biotin peroxidase immunohistochemistry. Hematoxylin and eosin staining of formalin-fixed paraffin-embedded human liver samples diagnosed as normal and hepatocellular carcinoma (HCC) are shown at the top of the image. High magnification images are shown in the inset. Scale bar shown is 100 *μ*m. These images were created by co-author Joon-Yong Chung at the National Cancer Institute, with permission privileges for their use.

The overall study design is shown in Fig 1. While PSMA is highly expressed in prostate cancers, it is also present on the tumor neovasculature, and in other malignancies, including up to 95% of HCC [4, 5]. Importantly, high PSMA expression was detected in HCC tissue whereas PSMA expression in normal liver tissue ranged from negative to weak (Fig 2). Functional imaging of HCC would be incredibly valuable, not only for diagnosis of *de novo* disease, but also for identifying recurrences. ^18^F-DCFPyL, a second generation PSMA imaging agent, has been approved by the FDA for use in men with prostate cancer, and we posit that it may serve as a useful functional imaging agent for patients with HCC [14–16].

### Objectives of the trial

The primary objective of the study is to assess the ability of ^18^F-DCFPyL PET/CT imaging to detect sites of hepatocellular carcinoma.

Secondary objectives include:

- To compare the ability of ^18^F-DCFPyL PET/CT to detect HCC with standard of care imaging (CT and/or MRI), and ^18^F-FDG PET/CT
- To assess the ability of ^18^F-DCFPyL PET/CT imaging to assess treatment response to local therapy

Exploratory Objectives include:

- Compare the distribution of ^18^F-DCFPyL uptake with standard of care CT and/or MRI.
- Compare focal abnormal ^18^F-DCFPyL uptake with focal abnormalities identified on ^18^F-FDG PET/CT.

- Compare PSMA expression with ^18^F-DCFPyL uptake.
- Compare tumor viability and metabolism determined by ^18^F-FDG PET imaging with ^18^F-DCFPyL uptake.
- Evaluate ^18^F-DCFPyL uptake with 5-years overall survival and progression free survival.
- Evaluate tumor biomarkers in blood, urine, and tissue samples.

## Methods

### Trial design

This is an open-label multi-site imaging study recruiting participants with suspected hepatocellular carcinoma (at least one detectable liver lesion visible on standard of care CT and/or MRI), who are eligible to undergo local treatment (e.g., surgical resection, radiofrequency ablation, microwave ablation, transarterial embolization (TAE), stereotactic body radiotherapy (SBRT)). Participants will be consented and enroll through the NIH Clinical Center. The protocol and its amendments have been approved by the Institutional Review Board (IRB) of the National Cancer Institute (NCI), National Institutes of Health (NIH), Bethesda, Maryland, USA. Clinical Trial registration: NIH IND#133631; NIH IRB #00080. ClinicalTrials.gov Identifier NCT05009979. IRB committee approval: 02/15/2022 (see full protocol and letter confirming funding in the supporting documents).

All participants will undergo a baseline ^18^F-DCFPyL PET/CT and ^18^F-FDG PET/CT. Each PET/CT scan will be performed approximately within 2 weeks, with minimum of a day apart from each other. A CT and/or MRI will be performed within 2 months of the ^18^F-DCFPyL PET/CT scan. Clinical scans performed in that time frame by local providers will also be acceptable.

Participants will undergo a biopsy prior to local therapy. For patients undergoing resection, the surgical specimen will suffice. The procedure may be performed at the NIH Clinical Center or at other participating sites.

Additional ^18^F-DCFPyL PET/CT imaging will be performed during the first routine follow-up (2–3 months post local therapy) for participants with a positive baseline ^18^F-DCFPyL-PET/CT (i.e. the presence of DCFPyL-avid tumor/s) and with a biopsy confirming HCC diagnosis. Subjects with negative tumor uptake at the baseline ^18^F-DCFPyL-PET/CT, and with a histopathology confirming HCC, will not undergo post-treatment imaging, but will remain in follow-up. Subjects with biopsies negative for HCC will be taken off protocol. Subjects with a histopathologically confirmed HCC will be followed by clinical chart review, phone-call, email follow-up or any other NIH-approved remote platform for tumor markers and radiologic evidence of recurrence over a 5-years period from the last ^18^F-DCFPyL PET/CT imaging. A set of imaging (^18^F-DCFPyL PET/CT, ^18^F-FDG PET/CT, CT/MRI) may be performed at the time of tumor recurrence per PI discretion. If participants undergo another treatment during the follow up period, a biopsy may be performed per PI discretion.

### Study population

#### Eligibility criteria

To be eligible to participate in this study, an individual must meet all of the following criteria:

1. Adult 18 years or older.
2. High radiological suspicion of hepatocellular carcinoma (LR4 or LR5 based on the most current version of liver reporting & data system (LI-RADS)) with at least one measurable lesion on standard imaging modality (CT and/or MRI).
3. Eligible for local therapies (included but not limited to surgical resection, stereotactic radiation therapy, trans-arterial chemo-, radio-, or bland embolization, microwave ablation, radiofrequency ablation).
4. Ability to take oral medication and be willing to adhere to the study intervention regimen.
5. Eastern Cooperative Oncology Group (ECOG) performance status ≤2.
6. Known human immunodeficiency virus (HIV)-infected individuals must be on effective anti-retroviral therapy with undetectable viral load within 6 months.
7. Known chronic hepatitis B virus (HBV) infected individuals, must be on suppressive therapy with undetectable viral load.
8. Individuals with a history of hepatitis C virus (HCV) infection must have been treated and cured.
9. The effects of ^18^F-DCFPyL (study drug) on the developing human fetus are unknown. For this reason and because this agent as well as other therapeutic agents used in this trial are known to be teratogenic, women of child-bearing potential and men must agree to use adequate contraception (hormonal or barrier method of birth control; abstinence) prior to study entry and for 2 months after each study PET/CT imaging. If a woman becomes pregnant or suspects she is pregnant while she or her partner is participating in this study, she will inform her treating physician immediately.
10. Ability of the subjects to understand and be willing to sign a written informed consent document.

An individual who meets any of the following criteria will be excluded from participation in this study:

1. History of allergic reactions attributed to compounds of similar chemical or biologic composition to ^18^F-DCFPyL or other agents used in study.
2. Uncontrolled intercurrent illness including, but not limited to, ongoing or active infection, symptomatic congestive heart failure, unstable angina pectoris, cardiac arrhythmia, or psychiatric illness/social situations that would limit compliance with study requirements.
3. Subjects with severe claustrophobia unresponsive to oral anxiolytics.
4. Other medical conditions deemed by the principal investigator (or associates) to make the subject unsafe/ineligible for protocol procedures.
5. Subjects weighing > 350 lbs (weight limit for scanner table), or unable to fit within the imaging gantry.
6. Serum creatinine > 2 times the upper limit of normal.
7. Pregnant women are excluded from this study because ^18^F-DCFPyL as well as other agents used in this trial are known or have the potential for teratogenic or abortifacient effects.

### Study settings

This is a multi-site, open-label, phase II trial (ClinicalTrials.gov Identifier: NCT050099). The patients’ recruitment will be performed at NCI, NIH, at the Veterans Affairs Medical Center, in Maryland and Washington DC, and at MedStar Georgetown University Hospital, Washington DC, USA.

### Interventions

#### Study procedure

All participants will undergo a baseline ^18^F-DCFPyL PET/CT at the Molecular Imaging Clinic, at NCI prior to any therapy. For participants with a positive baseline ^18^F-DCFPyL-PET/CT (i.e. the presence of DCFPyL-avid tumor/s), an additional ^18^F-DCFPyL PET/CT imaging will be performed during the first routine post local therapy follow-up to assess treatment effects.

#### PET imaging radiotracer

The radiolabeled ligand ^18^F-DCFPyL (NIH) will be used as the PET radiopharmaceutical. The target administered activity will be 9 mCi; dose variations will be in accordance with the Nuclear Regulatory Commission standard dose variation (i.e. 20%) permitted for diagnostic clinical studies. Due to potential unpredictable delays and the short half-life of F-18, the total dose of ^18^F-DCFPyL administered may be reduced at the discretion of the principal investigator or their designee. The administration site should be evaluated just before, during and after injection, to assess for extravasation and/or signs of local irritation. Because there is an unknown but potential risk for adverse events in nursing infants secondary to exposure of the mother to F-18, breastfeeding should be discontinued for 12 hours after administration of either ^18^F-DCFPyL.

#### PET/CT imaging protocol specifics

The ^18^F-DCFPyL PET/CT imaging will consist of the ^18^F-DCFPyL injection, followed by a 45-minutes dynamic PET/CT imaging of a single bed position (including the liver lesion in the field of view), and a static whole-body PET/CT imaging (top of head to mid-thighs) performed at 1 hour (+/- 10 minutes) post ^18^F-DCFPyL injection. The initial 45-minutes dynamic regional scan will be used to determine the kinetics of ^18^F-DCFPyL within the tumor as compared with normal liver and other adjacent normal background. Participants will be encouraged to hydrate and urinate frequently after the radiotracer administration.

Summary of scanning procedure:

1. IV placement.
2. Administration of ^18^F-DCFPyL; begin adverse events (AE) monitoring.
3. Dynamic PET/CT imaging of a single field-of-view (including the liver lesion) will be acquired for 45 minutes.
4. Subject asked to void.
5. 1 hour (+/- 10 minutes) following ^18^F-DCFPyL injection, static whole-body PET/CT imaging will be performed (~30–45 minutes in duration).
6. Follow-up AE query at 1–3 days post-injection.

#### PET/CT imaging analysis and interpretation

PET/CT images will be reviewed and analyzed using dedicated workstations and by using consensus clinical readouts in the Molecular Imaging Branch with access to all medical records. No blinded independent readers will be used.

^18^F-DCFPyL PET/CT will be used to identify positive suspected sites, which will be correlated with histopathology results. For lesions within the liver, a focal abnormal area of increased ^18^F-DCFPyL activity higher than the surrounding liver uptake, with a maximum standardized uptake value (SUV_max_) greater than 1.2 times the normal liver SUV mean will be considered positive. For lesions outside the liver, a positive lesion will be defined as focal abnormal uptake higher than the blood pool, or the surrounding soft tissue or normal organ background. Pathological anatomic imaging lacking DCFPyL uptake would indicate a type of tumor that fails to express PSMA. The performance of ^18^F-DCFPyL PET/CT is assessed by the positive predictive value, which is defined as the proportion of histopathology positive lesions.

Images will be analyzed by identifying, recording, and measuring measurable target lesions. A segmentation tool will be applied to generate a semi-automated volume of interest (VOI) within the target lesion(s). SUV_max_ and tumor volumes will be extracted from the generated target lesion(s) VOIs. All the other lesions or sites of disease including any measurable lesions will be identified as non-target lesions and will be recorded.

To determine whether uptake of ^18^F-DCFPyL correlates with response to therapy, target tumor(s) imaging PET parameters, including SUV_max_ and tumor volume will be compared between responders and non-responders. Participants’ treatment response will be based on RECIST 1.1. criteria and clinical assessment.

### Biological specimens

#### Immunohistochemistry and PSMA staining on tissue samples

Specimen (1 core) will undergo routine formalin fixation and paraffin embedding, immunohistochemistry, tumor diagnosis, and molecular evaluation in the NCI Laboratory of Pathology. PSMA staining will also be conducted. The specimen results will be correlated with ^18^F-DCFPyL PET/CT and standard CT and/or MRI imaging results.

#### HCC blood biomarker studies

Alpha-fetoprotein (AFP), carcinoembryonic antigen (CEA), carbohydrate 19.9 (CA19.9) and liver function tests (alkaline phosphatase (ALP), aspartate transaminase (AST), alanine transaminase (ALT), total bilirubin, direct bilirubin) will be performed within 30 days of each ^18^F-DCFPyL PET/CT imaging.

#### Analysis of extracellular vesicles and particles

Blood and urine samples will be used for analysis of extracellular vesicles and particles (EVPs). Specifically, we are most interested in measuring the concentration and composition of the EVPs that are PSMA^+^ across the samples, as related to PSMA detection on imaging and in response to treatment. We have identified robust methods for detecting and isolating PSMA^+^ EVPs, and then evaluating co-expressed proteins and nucleic acids associated with those EVPs. EVPs in general will be processed and evaluated according to our published protocols (nano.ccr.cancer.gov), with PSMA^+^ EVPs enriched by immunoaffinity from cell-free blood and urine samples. Our hypothesis is that the molecular cargo and composition of the PSMA+ EVPs in liquid biopsies (i.e., accessible in blood and urine samples) may correlate with 1) imaging extent of PSMA detection, 2) additional histopathologic features identified in the targeted biopsies in the study, and 3) the biological/clinical course of PSMA^+^ foci, followed over time.

The following analysis will be performed:

1. Isolation of EVPs will be performed with size exclusion and affinity chromatography.
2. Enumeration and characterization of EVPs and their composition will be performed with nanoparticle tracking analysis, resistive pulse sensing, and flow cytometry to determine whether volume of PSMA+ tissue on imaging correlates with concentration of PSMA+ EVPs in blood and/or urine. Routine laboratory assays for protein/DNA/RNA quantification will also be performed.
3. Stranded RNA sequencing and DNA methylomics will be compared between pooled PSMA^+^ (or other surface marker positive) EVP vs PSMA^-^ (marker negative) isolates to identify distinctive cargo signatures that correlate with disease extent, progression, or other clinical/histopathologic correlates.

#### Next generation sequencing from tissue and blood samples

In order to address the exploratory goal of characterizing predictive biomarkers of response, including but not limited to genomic, transcriptomic (RNA Seq), and/or epigenomic assessments of tumor tissue may be performed.

### Outcomes

#### Primary endpoint measures

The point estimates and 95% confidence intervals of the positive predictive value of ^18^F-DCFPyL PET/CT will be reported in which the confidence limits are the 2.5th and 97.5th percentile of the 2000 bootstrap samples obtained by random sample without replacement at the participant level to account for inter-lesion correlation.

#### Secondary endpoints

- The lesion level sensitivity, specificity and positive predictive value of ^18^F-DCFPyL PET/CT and CT/MRI will be calculated and compared. The confidence interval for each estimate will be obtained from the bootstrap samples and the difference in the estimates between the imaging modalities will be compared by the Wald test with the standard error calculated from the bootstrap samples.
- For ^18^F-DCFPyL PET/CT positive participants who undergo local treatment for HCC, change in ^18^F-DCFPyL PET/CT uptake between pre- and post-treatment of tumor or tumor bed will be compared by paired Wilcoxon test.

#### Exploratory endpoints

- The uptake of ^18^F-DCFPyL PET/CT will be correlated with CT/MRI grade by Kendall’s tau-b correlation.
- The uptake of ^18^F-DCFPyL PET/CT will be correlated with tumor tissue histological PSMA expression by Kendall’s tau-b correlation.
- The uptake of ^18^F-DCFPyL PET/CT measured by maximum Standard Uptake Value (SUV) will be correlated with ^18^F-FDG uptake by Spearman rank correlation. These SUV uptake measurements will also be correlated with PSMA expression as determined by standard histological assessment of tumor tissue.
- Uptake of ^18^F-DCFPyL PET/CT with be correlated with 5 year progression free survival (via scans collected from local providers during routine follow up) and 5 year overall survival by Cox regression analysis.
- Descriptive statistics will be used to summarize tumor biomarkers in blood and tissue samples.

### Sample size

Sample size was calculated such that the limits of the 95% expected confidence interval to the true lesion level positive predicted value is less than 15%. The lesion level positive predictive value is the proportion of biopsy positive lesions. Assume 80% of the participants enrolled to the study each have 1–2 lesions identified by ^18^F-DCFPyL-PET/CT with 0.2 modest inter-lesion correlation, and the positive predictive value is 70%. A sample size of 40 evaluable participants accrued to the study produces a two-sided 95% confidence interval with the distance from the positive predictive value to the limits equal to 0.148. An evaluable participant is defined as a participant who completes all required study procedures. To account for non-evaluable participants, the accrual ceiling will be set to 50.

### Recruitment

This protocol may be abstracted into a plain language announcement posted on NIH websites and on NIH social media platforms. Participants will also be identified via internal referrals from each participating site institution, and outside referrals.

### Interim analysis and early stopping rule

To avoid radiation exposure and excessive expense, if ^18^F-DCFPyL PET/CT is not able to successful identify HCC lesions, we will implement the following interim futility analysis and early stopping rule. We plan to stop the protocol if the first 5 participants with lesions greater than 1 cm have negative DCFPyL uptake (defined as tumor uptake less than adjacent background soft tissue, or less than blood pool for lymph nodes).

### Participant timeline

**Fig 3:**
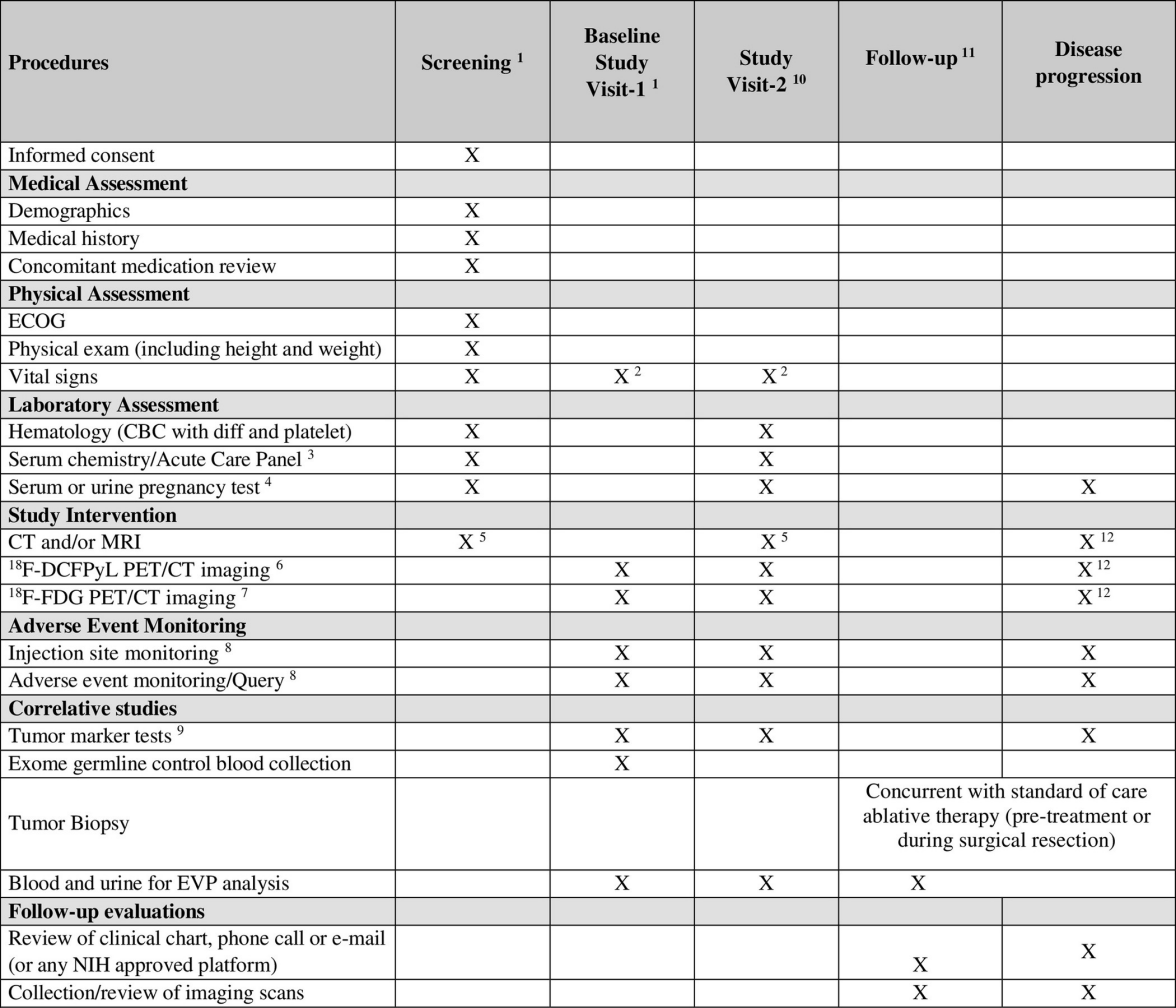
Study calendar. Table numbers’ details: 1. Performed within 30 days prior to administration of ^18^F-DCFPyL unless otherwise indicated. Screening procedures such as medical assessment may be performed remotely via any NIH approved platforms. 2. Vital signs will be taken prior to injection of ^18^F-DCFPyL and following completion of the final PET/CT scan (+ 15 minutes). 3. Acute care panel: sodium, potassium, chloride, total CO2, creatinine, glucose, urea nitrogen, eGFR 4. For female participants of childbearing age (in the absence of prior hysterectomy). Pregnancy tests may be performed as clinically indicated prior to each scan. 5. CT (Chest/Abdomen/Pelvis) and/or MRI (Abdomen) will be performed within 2 months of each ^18^F-DCFPyL PET/CT. Imaging may be performed at certified outside facility and provided to study team. 6. Subjects will undergo ^18^F-DCFPyL injection and a dynamic PET/CT. Approximately 1 hour (+/- 10 minutes) post ^18^F-DCFPyL injection, a static PET/CT imaging performed. Refer to section 3.2.2 for additional information regarding the scanning procedure. 7. ^18^F-FDG PET/CT will be performed within approximately 2 weeks before or after each ^18^F-DCFPyL scan. Each PET/CT imaging must be approximately a day apart. The order obtained for ^18^F-DCFPyL PET/CT and ^18^F-FDG PET/CT does not matter. 8. Event monitoring will be done at the time of injection, and 1 hour post injection. All subjects will be contacted by phone at ~1–3 business days post-injection and will be asked non-leading questions regarding symptoms. At the investigator’s discretion, subjects with safety concerns noted during the post injection period may remain at the site or be asked to return to the site to undergo further safety assessments at the 1–3 business day follow-up time point. 9. Tumor markers include Alpha-fetoprotein, carcinoembryonic antigen, carbohydrate antigen 19.9 (CA19.9) and liver function tests (Alkaline Phosphatase, ALT, AST, Total Bilirubin, Direct Bilirubin). Refer to section 5.1 for more details. 10. Only participants with a positive baseline ^18^F-DCFPyL-PET/CT scan (i.e. with the presence of DCFPyL-avid tumor/s) and a biopsy confirming HCC diagnosis will undergo visit 2 for a second ^18^F-DCFPyL PET/CT (during routine treatment follow up, typically within 4–8 weeks). 11. Follow-up will be performed every 3 months after the last ^18^F-DCFPyL scan (or after therapy for participants with negative baseline ^18^F-DCFPyL PET/CT and biopsy confirming HCC) for 2 years, and yearly afterwards for an additional 3 years. 12. If tumor recurrence occurs during the follow-up period, an ^18^F-DCFPyL PET/CT, ^18^F-FDG PET/CT, CT/MRI and biopsy may be performed any time after recurrence at PI discretion. Biopsy would be performed concurrently with treatment if the latter is performed.

Referring physicians will be educated on the study goals and logistics in order to recruit potential eligible and interested patients. The patients’ decision to participate will be entirely voluntary. Eligible patients who decide not to participate will be offered all other standard of care approaches. Patients will be consented either in person or over the phone. Time schedule of enrolment, interventions, assessments, and visits are included in Fig 3.

### Data management

Samples will be ordered in the Clinical Research Information System (CRIS) and tracked through a Clinical Trial Data Management system. Should a CRIS screen not be available, the CRIS downtime procedures will be followed. Samples will not be sent outside NIH without appropriate approvals and/or agreements, if required. The investigators at each site will be responsible for overseeing entry of data into a 21 CFR Part 11 compliant data capture system provided by the NCI Clinical Cancer Research and ensuring data accuracy, consistency, and timeliness. Data should be received by the coordinating institution at least quarterly.

### Statistical methods

#### Analysis of the primary endpoint

The point estimates and 95% confidence intervals of the positive predictive value of ^18^F-DCFPyL PET/CT will be reported in which the confidence limits are the 2.5^th^ and 97.5^th^ percentile of the 2000 bootstrap samples obtained by random sample without replacement at the participant level to account for inter-lesion correlation.

#### Analysis of the secondary endpoints

- The lesion level sensitivity, specificity, and positive predictive value of ^18^F-DCFPyL PET/CT and CT/MRI will be calculated and compared. The confidence interval for each estimate will be obtained from the bootstrap samples and the difference in the estimates between the imaging modalities will be compared by the Wald test with the standard error calculated from the bootstrap samples.
- For ^18^F-DCFPyL PET/CT positive participants who undergo local treatment for HCC, change in ^18^F-DCFPyL PET/CT uptake between pre- and post-treatment of the tumor or tumor bed will be compared by paired Wilcoxon test.

#### Analysis of the exploratory endpoints

- The uptake of ^18^F-DCFPyL PET/CT will be correlated with CT/MRI grade by Kendall’s tau-b correlation.
- The uptake of ^18^F-DCFPyL PET/CT will be correlated with tumor tissue histological PSMA expression by Kendall’s tau-b correlation.
- The uptake of ^18^F-DCFPyL PET/CT (measured by SUV) will be correlated with ^18^F-FDG uptake by Spearman rank correlation. These SUV uptake measurements will also be correlated with PSMA expression as determined by standard histological assessment of tumor tissue.
- Uptake of ^18^F-DCFPyL PET/CT will be correlated with 5-year progression free survival (via scans collected from local providers during routine follow up) and 5-year overall survival by Cox regression analysis.
- Kinetic parameters of ^18^F-DCFPyL dynamic imaging will be correlated with physiologically-based pharmacokinetic (PBPK) modeling based on tissue microenvironment characteristics at baseline.
- Descriptive statistics will be used to summarize tumor biomarkers in blood and tissue samples.

### Data monitoring

The clinical research team will meet on a regular basis (weekly) when participants are being actively treated on the trial to discuss each participant. All data will be collected in a timely manner and reviewed by the principal investigator. Events meeting requirements for expedited reporting will be submitted within the appropriate timelines. The principal investigator will review adverse events on each participant to ensure safety and data accuracy. The principal investigator will personally conduct or supervise the investigation and provide appropriate delegation of responsibilities to other members of the research staff.

Each clinical site will perform internal quality management of study conduct, data and biological specimen collection, documentation and completion. An individualized quality management plan will be developed to describe a site’s quality management. Quality control (QC) procedures will be implemented beginning with the data entry system and data QC checks that will be run on the database will be generated. Any missing data or data anomalies will be communicated to the site(s) for clarification/resolution. Following written Standard Operating Procedures (SOPs), the monitors will verify that the clinical trial is conducted, and data are generated and biological specimens are collected, documented (recorded), and reported in compliance with the protocol, International Conference on Harmonisation Good Clinical Practice (ICH GCP), and applicable regulatory requirements (e.g. Good Laboratory Practices (GLP), Good Manufacturing Practices (GMP)). The investigational site will provide direct access to all trial related sites, source data/documents, and reports for the purpose of monitoring and auditing by the sponsor, and inspection by local and regulatory authorities.

### Confidentiality

Participant confidentiality and privacy is strictly held in trust by the participating investigators and staff. This confidentiality is extended to cover testing of biological samples and genetic tests in addition to the clinical information relating to participants. All research activities will be conducted in as private a setting as possible. The study participant’s contact information will be securely stored at the/each clinical site for internal use during the study. At the end of the study, all records will continue to be kept in a secure location for as long a period as dictated by the reviewing IRB or Institutional policies. Study participant research data, which is for purposes of statistical analysis and scientific reporting, will be transmitted to and stored at the NCI Clinical Cancer Research (CCR). This will not include the participant’s contact or identifying information. Rather, individual participants and their research data will be identified by a unique study identification number. The study data entry and study management systems used by the clinical site(s) and by NCI CCR research staff will be secured and password protected. At the end of the study, all study databases will be archived at the NIH. To further protect the privacy of study participants, a Certificate of Confidentiality has been issued by the National Institutes of Health (NIH).

### Access to data and materials

The principal investigator and the Molecular imaging Branch staff at the NCI, NIH will have full access to all interim and final data results of the study through the CRIS database. All data generated and/or analyzed during this study will be available in a future publication.

### Dissemination of results

The results of this trial will be submitted for publication in national or international peer-reviewed journals with all collaborators acknowledged. Results will also be disseminated through conference presentations. It is expected that several publications will originate from this protocol, addressing the aims as mentioned above.

## Discussion

While conventional imaging and the use of LIRADS represents the gold standard for the initial diagnosis of HCC, challenges remain in the recurrent setting. A functional imaging agent that can be used in patients who have received local treatments could significantly help in clinical decision-making (e.g. surveillance, treatment planning). Furthermore, such an agent could provide additional utility for *de novo* disease, potentially differentiating between high risk versus low risk LR3 or LR4 lesions. While bespoke tumor-selective functional imaging agents for HCC remain years away, repurposing existing agents is immediately evaluable.

PSMA is highly expressed in prostate cancers, yet is also present on the tumor neovasculature, and in other malignancies, including HCC. ^18^F-DCFPyL, a second generation ^18^F-labeled PSMA imaging agent, has been approved for use in men with prostate cancer by the FDA, and we posit that it may serve as a useful functional imaging agent for patients with HCC.

Our phase II prospective multisite trial designed to determine whether PSMA PET/CT imaging can detect tumor sites and assess treatment response in patients with histopathologically-confirmed HCC receiving local therapy. In conjunction, we will explore several correlative studies to help identify biomarkers associated with response and recurrence.

If successful, results from our trial may yield a strategy to identify, characterize, and monitor treatment response for HCC with higher fidelity than conventional methods in the *de novo* and recurrent settings. Furthermore, findings could open the possibility of tumor-selective therapy with PSMA-directed therapeutics, including the beta and alpha emitting radiopharmaceuticals administered intravenously or transarterially [30, 31]. Notably, transarterial administration of radiopharmaceuticals with ^90^Y-labeled microspheres is FDA-approved for patients with unresectable HCC ≤ 8 cm [32], and personalized dosimetry with this modality appears to augment treatment response [33]. PSMA-selective radiopharmaceuticals could be similarly implemented in locally advanced HCC, and, unlike other liver directed therapies could also be administered intravenously in the metastatic setting as well. There is a precedent for systemic radiopharmaceutical therapy for HCC with the CD147-targeting ^131^I-metuximab that has shown promising efficacy in a phase II study, warranting confirmatory definitive phase III studies [34]. Similarly, ^131^I-metuximab has been successfully used combined with TACE for unresectable HCC with improved overall survival [35]. This approach may complement existing systemic therapy options [33, 34, 36, 37].

In summary, functional imaging could facilitate diagnosis and surveillance of HCC for clinical decision-making. Given PSMA-expression in HCC and its vasculature, ^18^F-DCFPyL could help fill this unmet need and our trial (NCT05009979), which is now recruiting, along with others in Mayo Clinic NCT04310540 and NCT04762888), in Australia (NCT05095519) and China (NCT05006326), aims to answer this question.

## Supporting information

S1 Protocol Full protocol. (PDF)

S1 File (PDF)

S2 File Confirmation letter of funding. (PDF)

S1 Checklist SPIRIT 2013 checklist: Recommended items to address in a clinical trial protocol and related documents*. (PDF)
