# Supplementary material for: Functional Imaging of Liver Cancer (FLIC): Study protocol of a phase 2 trial of 18F-DCFPyL PET/CT imaging for patients with hepatocellular carcinoma
Source: PLoS One. 2022 Nov 11;17(11):e0277407. doi: 10.1371/journal.pone.0277407 (PMC9651549; doi:10.1371/journal.pone.0277407)
Supplement: S1 Protocol — (PDF) [file pone.0277407.s001.pdf]

Abbreviated Title: 18F-DCFPyL in HCC
NIH Protocol #: 000080
NIH Protocol Version Date: 05/06/2022
NCI Supplement Version Date: 05/06/2022
NCT Number: NCT05009979

Title: ^{18}F-DCFPyL PET/CT in Hepatocellular Carcinoma

NCI Principal Investigator: Freddy E. Escorcia, MD, PhD
Molecular Imaging Branch
National Cancer Institute
Building 10, Rm 1B55
9000 Rockville Pike
Bethesda, MD 20892
Phone: 240-858-3062
E-mail: freddy.escorcia@nih.gov

Investigational Agents:

Drug Name:	^{18}F-DCFPyL	^{18}F-FDG
IND Number:	133631	133631
Sponsor:	NCI CCR	NCI CCR
Manufacturer:	PET department, NIH	Commercial
Supplier:	PET department, NIH	MIB

Coordinating Center: NCI, CCR

TABLE OF CONTENTS

TABLE OF CONTENTS 2
1 NUMBER OF PARTICIPANTS TO BE SEEN AT NIH 3
2 NIH REGISTRATION INFORMATION 3
2.1 Participant Registration and Status Update Procedures 3
2.2 Cost and Compensation 3
2.2.1 Costs 3
2.2.2 Compensation 3
2.2.3 Reimbursement 3
3 CORRELATIVE STUDIES 3
3.1 Sample Storage, Tracking and Disposition 3
3.1.1 Laboratory of Pathology 3
3.1.2 Dr. Wang Laboratory 4
3.1.3 Dr. Jennifer Jones Laboratory 4
3.1.4 Blood Processing Core (Laboratory of Dr. William Figg) 4
3.1.5 CCR Sequencing Facility 5
3.2 Samples for Genetic/Genomic Analysis 6
3.2.1 Genetic Counseling 6
4 NCI CLINICAL DIRECTOR REPORTING 6
5 CONSENT DOCUMENTATION WHEN ELECTRONIC CONSENT DOCUMENT USED AT NIH 6

1 NUMBER OF PARTICIPANTS TO BE SEEN AT NIH

All 50 participants will be enrolled at the NIH. Some participants may have biopsies performed at the participating site.

2 NIH REGISTRATION INFORMATION

2.1 Participant Registration and Status Update Procedures

Registration and status updates (e.g., when a participant is taken off protocol therapy and when a participant is taken off-study) will take place per CCR SOP ADCR-2, CCR Participant Registration & Status Updates found here.

2.2 Cost and Compensation

2.2.1 Costs

Please refer to section **3.6.1** of main protocol.

2.2.2 Compensation

Please refer to section **3.6.2** of main protocol.

2.2.3 Reimbursement

The NCI will cover the costs of some expenses associated with protocol participation. Some of these costs may be paid directly by the NIH and some may be reimbursed to the participant/guardian as appropriate. The amount and form of these payments are determined by the NCI Travel and Lodging Reimbursement Policy.

3 CORRELATIVE STUDIES FOR RESEARCH

3.1 Sample Storage, Tracking and Disposition

Samples will be ordered in CRIS and tracked through a Clinical Trial Data Management system. Should a CRIS screen not be available, the CRIS downtime procedures will be followed. Samples will not be sent outside NIH without appropriate approvals and/or agreements, if required.

3.1.1 Laboratory of Pathology

3.1.1.1 Clinical Tissue Samples

Tissues designated for clinical diagnostics are transported to the Laboratory of Pathology (LP) where they are examined grossly and relevant portions are fixed, embedded in paraffin and sectioned and stained for diagnostic interpretation. Unutilized excess tissue that is not placed in paraffin blocks is stored in formalin for up to three months, in accordance with College of American Pathologists/Joint Commission on Accreditation of Healthcare Organizations (CAP/JCAHO) guidelines, and then discarded. Following completion of the diagnostic workup, the slides and tissue blocks are stored indefinitely in the LP's clinical archives. All specimens are catalogued and retrieved utilizing the clinical laboratory information systems, in accordance with

CAP/JCAHO regulations. The use of any stored specimens for research purposes is only allowed when the appropriate IRB approval has been obtained. In some cases, this approval has been obtained via the original protocol on which the participant was enrolled.

3.1.1.2 Research Tissue and Blood Specimen (COMPASS Program)

All FFPE tissues that will undergo molecular pathology testing should be submitted through Surgical Pathology. Blood specimen should be delivered to room 3S247 in Building 10 (samples will be accepted from 7:30 AM to 4:30 PM Monday to Friday). Sample storage, tracking and disposition procedures will follow laboratory of pathology standard practice (section **3.1.1**).

3.1.2 Dr. Wang Laboratory

Surgical resected tumor tissue or needle biopsy core will be received after processing. Tumor tissue will be cut into small pieces and dissociated into single cells. For surgical resected tissue, part of tissue will be fixed for histological staining and excess tissue frozen down in liquid nitrogen. Each collection point may have multiple vials of cells frozen down, depending on the total number of cells. For those time points that have multiple vials of enough cells, different sequencing (e.g. single cell RNA-seq and ATAC-seq) could be done in parallel. The single cell and tissue storage will be recorded in Labmatrix. Records are updated upon sample disposition.

3.1.3 Dr. Jennifer Jones Laboratory

Serum and urine EVP samples will be stored in Dr. Figg's Biospecimen Processing Biorepository and will be analyzed in the Jones Laboratory. Samples will be processed in Building 10/B1B51/B1B53 and the LP COMPASS facility, with samples maintained during processing in rooms that are locked by badge access to only authorized personnel.

3.1.4 Blood Processing Core (Laboratory of Dr. William Figg)

3.1.4.1 Sample Collection

Remaining tissue samples will be obtained from the laboratory of pathology and stored in Dr. Figg's lab.

Please e-mail NCIBloodcore@mail.nih.gov at least 24 hours before transporting samples (the Friday before is preferred).

For sample pickup, page 102-11964.

For immediate help, call 240-760-6180 (main blood processing core number) or, if no answer, 240-760-6190 (main clinical pharmacology lab number).

For questions regarding sample processing, contact NCIBloodcore@mail.nih.gov

The samples will be processed, barcoded, and stored in Dr. Figg's lab until requested by the investigator.

3.1.4.2 Sample Data Collection

All samples sent to the Blood Processing Core (BPC) will be barcoded, with data entered and stored in Labmatrix utilized by the BPC. This is a secure program, with access to Labmatrix limited to defined Figg lab personnel, who are issued individual user accounts. Installation of Labmatrix is limited to computers specified by Dr. Figg. These computers all have a password restricted login screen.

Labmatrix creates a unique barcode ID for every sample and sample box, which cannot be traced back to participants without Labmatrix access. The data recorded for each sample includes the patient ID, name, trial name/protocol number, time drawn, cycle time point, dose, material type, as well as box and freezer location. Participant demographics associated with the Clinical Center patient number are provided in the system. For each sample, there are notes associated with the processing method (delay in sample processing, storage conditions on the ward, etc.).

3.1.4.3 Sample Storage and Destruction

Barcoded samples are stored in barcoded boxes in a locked freezer at either -20 or -80°C according to stability requirements. These freezers are located onsite in the BPC and offsite at NCI Frederick Central Repository Services in Frederick, MD. Visitors to the laboratory are required to be accompanied by laboratory staff at all times.

Access to stored clinical samples is restricted. Samples will be stored until requested by a researcher named on the protocol. All requests are monitored and tracked in Labmatrix. All researchers are required to sign a form stating that the samples are only to be used for research purposes associated with this trial (as per the IRB approved protocol) and that any unused samples must be returned to the BPC. It is the responsibility of the NCI Principal Investigator to ensure that the samples requested are being used in a manner consistent with IRB approval.

Following completion of this study, samples will remain in storage as detailed above. Access to these samples will only be granted following IRB approval of an additional protocol, granting the rights to use the material.

If, at any time, a participant withdraws from the study and does not wish for their existing samples to be utilized, the individual must provide a written request. Following receipt of this request, the samples will be destroyed or returned to the participant, if so requested. The PI will record any loss or unanticipated destruction of samples as a deviation. Reporting will be per the requirements of section **7.2** of the main protocol.

Sample barcodes are linked to participant demographics and limited clinical information. This information will only be provided to investigators listed on this protocol, via registered use of the Labmatrix. It is critical that the sample remains linked to participant information such as race, age, dates of diagnosis and death, and histological information about the tumor, in order to correlate genotype with these variables.

3.1.5 CCR Sequencing Facility

RNA samples should be transported in dry ice. The names on the tubes are checked against the sample manifest and stored in a -80°C freezer. Sample data is entered in LIMS once samples have undergone a quality check (QC) according to established lab protocols. A sample QC report is generated and sent to the PI. LIMS labels are generated from the QC data and used to re-label the sample tubes. Once the QC is complete, samples are stored into the library construction box in the -80°C freezer.

The libraries are generated either manually or using automation following the established library prep protocol. Any leftover RNA is stored in the -80°C freezer. If the libraries pass QC, a dilution of the library is passed on for qPCR and sequencing, and the library is stored along with the original RNA sample in the same box at -80°C. Each step is documented in a tracking sheet and LIMS.

The library dilutions are all stored at -30°C until sequencing is completed. Once the sequencing data is delivered, RNA samples and libraries are sent back to the PI.

3.2 Samples for Genetic/Genomic Analysis

3.2.1 Genetic Counseling

If the research findings are verified in the CLIA certified lab, the subject will be offered the opportunity to come to NIH (at our expense) to have genetic education and counseling with the NCI Genetics Branch to explain this result. If the subject does not want to come to NIH, a referral to a local genetic healthcare provider will be provided (at their expense). This is the only time during the course of the study that incidental findings will be returned. No interrogations regarding clinically actionable findings will be made after the primary analysis.

4 NCI CLINICAL DIRECTOR REPORTING

Problems expeditiously reported to the OHSRP in iRIS will also be reported to the NCI Clinical Director. A separate submission is not necessary as reports in iRIS will be available to the Clinical Director.

In addition to those reports, all deaths that occur within 30 days after receiving a research intervention should be reported via email to the Clinical Director unless they are due to progressive disease.

To report these deaths, please send an email describing the circumstances of the death to the Clinical Director/designee at NCICCRQA@mail.nih.gov within one business day of learning of the death.

5 CONSENT DOCUMENTATION WHEN ELECTRONIC CONSENT DOCUMENT USED AT NIH

Manual (non-electronic) signature on electronic document:

When a manual signature on an electronic document is used for the documentation of consent at the NIH Clinical Center, this study will use the following to obtain the required signatures:

- Adobe platform (which is not 21 CFR Part 11 compliant); or,
- iMedConsent platform (which is 21 CFR Part 11 compliant)

During the consent process, participants and investigators will view individual copies of the approved consent document on screens at their respective locations (if remote consent); the same screen may be used when in the same location but is not required.

Both the investigator and the participant will sign the document using a finger, stylus or mouse.

Note: Refer to the CCR SOP PM-2, Obtaining and Documenting the Informed Consent Process for additional information (e.g., verification of participant identity when obtaining consent remotely) found here.

Abbreviated Title: 18F-DCFPyL in HCC
NIH Protocol #: 000080
Version Date: 05/06/22022
NCT: NCT05009979

Title: ^{18}F-DCFPyL PET/CT in Hepatocellular Carcinoma

NIH Principal Investigator: Freddy E. Escorcia, MD, PhD
Molecular Imaging Branch
National Cancer Institute
Building 10, Rm 1B55
9000 Rockville Pike
Bethesda, MD 20892
Phone: 240-858-3062
E-mail: freddy.escorcia@nih.gov

Investigational Agents:

Drug Name:	**^{18}F-DCFPyL**	**^{18}F-FDG**
IND Number:	133631	133631
Sponsor:	NCI CCR	NCI CCR
Manufacturer:	PET department, NIH	Commercial

Commercial Imaging: CT scan, MRI
Coordinating Center: NCI, CCR

PRÉCIS

Background:

- Prostate specific membrane antigen is overexpressed in high-grade tumors, and increases when de-differentiation, metastatic or hormone-refractory disease occur, making the expression level a prognostic factor for disease outcome.
- It has been shown that PSMA can be expressed not only on prostate cancer cells, but also on cell lines of other malignancies, as well as tumor endothelium.
- A recent publication reported that nearly 95% of hepatocellular carcinoma (HCC) stained positive for PSMA in the tumor vasculature. Research suggests that the process of endothelial cell recruitment to HCC occurs early and throughout the process of hepatic tumorigenesis, making an endothelial cell tracer an ideal marker to detect early disease.
- ^{18}F-DCFPyL, a second generation PSMA PET agent, binds with high affinity to PSMA yet clears rapidly from the blood pool and thus, whole-body PET imaging with this agent, may provide a new tool in staging high risk cancers and detecting recurrent disease.
- We propose to expand our clinical work using ^{18}F-DCFPyL, and evaluate its usefulness for detecting sites of hepatocellular carcinoma.

Objective:

- To assess the ability of ^{18}F-DCFPyL PET/CT imaging to detect sites of hepatocellular carcinoma

Eligibility:

- Participants ≥ 18 years old
- High radiological suspicion of hepatocellular carcinoma (HCC) with at least one measurable lesion on standard imaging modality (CT and/or MRI)
- Eastern Cooperative Oncology Group (ECOG) Performance score of 0 to 2

Design:

- This is a multi-site imaging study enrolling participants with suspected hepatocellular carcinoma. The accrual ceiling is set to 50 participants.
- All participants will undergo a baseline ^{18}F-DCFPyL PET/CT scan. A standard of care CT and/or MRI will be performed within 2 months of the ^{18}F-DCFPyL PET/CT. Participants will be also scanned with an ^{18}F-FDG PET/CT imaging within approximately 2 weeks of the ^{18}F-DCFPyL PET/CT imaging.
- Participants will be scheduled to undergo a biopsy prior to or during standard of care local treatment for HCC (e.g., resection, radiofrequency ablation, microwave ablation, transarterial embolization (TAE), stereotactic body radiotherapy (SBRT)).
- Participants with a baseline positive ^{18}F-DCFPyL-PET/CT imaging (i.e. with the presence of DCFPyL-avid tumor/s) and biopsy confirming HCC diagnosis will undergo a post-treatment ^{18}F-DCFPyL PET/CT imaging during the first routine follow-up period, typically within 4-8 weeks. Subjects with negative tumor uptake at baseline ^{18}F-DCFPyL-PET/CT will not be re-scanned post-treatment but will remain in follow-up.
- Participants with a positive HCC biopsy will be followed for 5 years to assess progression free survival.

TABLE OF CONTENTS

PRÉCIS 2
TABLE OF CONTENTS 3
STATEMENT OF COMPLIANCE 7
1 INTRODUCTION 8
1.1 Study Objectives 8
1.1.1 Primary Objective 8
1.1.2 Secondary Objectives 8
1.1.3 Exploratory Objectives 8
1.2 Background and Rationale 8
1.2.1 ^{18}F-DCFPyL 8
1.2.2 Toxicity and Pharmacology 10
1.2.3 Safety 11
1.3 Study Rationale 11
1.3.1 PSMA Expression in Human Cancers 11
1.3.2 Hepatocellular Carcinoma (HCC) 12
1.3.3 Hepatocellular Carcinoma and PSMA 12
1.3.4 ^{18}F-DCFPyL in HCC 14
2 ELIGIBILITY ASSESSMENT AND ENROLLMENT 14
2.1 Eligibility Criteria 14
2.1.1 Inclusion Criteria 14
2.1.2 Exclusion Criteria 15
2.2 Recruitment Strategies 15
2.3 Screening Evaluation 15
2.3.1 Screening activities performed prior to obtaining informed consent 15
2.3.2 Screening activities performed after a consent for screening has been signed 15
2.4 Screen Failures 16
2.5 Intervention Assignment Procedures 16
3 STUDY IMPLEMENTATION 17
3.1 Study Design 17
3.2 Agent Administration 17
3.2.1 ^{18}F-DCFPyL administration 17

3.2.2 ^{18}F-DCFPyL PET/CT imaging 18
3.2.3 ^{18}F-FDG PET/CT imaging 18
3.3 Study Calendar 19
3.4 Surgical Guidelines 22
3.5 Cost and Compensation 22
3.5.1 Costs 22
3.5.2 Compensation 22
3.6 Criteria for Removal from Protocol intervention and Off Study Criteria 22
3.6.1 Criteria for removal from protocol intervention 22
3.6.2 Off-Study Criteria 22
3.6.3 Lost to Follow-up 22
4 CONCOMITANT MEDICATIONS/MEASURES 23
5 CORRELATIVE STUDIES FOR RESEARCH 24
5.1 Biospecimen Collection 24
5.1.1 IHC and PSMA Staining on Tissue Samples 26
5.1.2 HCC Blood Biomarker Studies 26
5.1.3 Analysis of Extracellular Vesicles and Particles (EVPs) 26
5.1.4 Next Generation Sequencing from Tissue and Blood Samples 26
5.2 Instructions for participating sites to ship tumor samples 27
5.3 Sample Storage, Tracking and Disposition 27
5.4 Samples for Genetic/Genomic Analysis 27
5.4.1 Description of the scope of genetic/genomic analysis 27
5.4.2 Management of Results 27
5.4.3 Genetic Counseling 27
6 DATA COLLECTION AND EVALUATION 28
6.1 Data Collection 28
6.2 Data Sharing Plans 28
6.2.1 Human Data Sharing Plan 28
6.2.2 Genomic Data Sharing Plan 29
6.3 Toxicity Criteria 29
7 NIH REPORTING REQUIREMENTS / DATA AND SAFETY MONITORING PLAN 30
7.1 Definitions 30

7.2 OHSRP Office of Compliance and Training / IRB Reporting 30
7.2.1 Expedited Reporting 30
7.2.2 IRB Requirements for PI Reporting at Continuing Review 30
7.3 NCI Guidance for Reporting Expedited Adverse Events for Multi-Center Trials 30
7.4 NIH Required Data and Safety Monitoring Plan 30
7.4.1 Principal Investigator/Research Team 30
8 SPONSOR PROTOCOL/SAFETY REPORTING 31
8.1 Definitions 31
8.1.1 Adverse Event 31
8.1.2 Serious Adverse Event (SAE) 31
8.1.3 Life-threatening 31
8.1.4 Severity 32
8.1.5 Relationship to Study Product 32
8.2 Assessment of Safety Events 32
8.3 Reporting of Serious Adverse Events 32
8.4 Waiver of expedited reporting to CCR 33
8.5 Reporting Pregnancy 33
8.5.1 Maternal Exposure 33
8.5.2 Paternal Exposure 33
8.6 Regulatory Reporting For Studies Conducted Under CCR Sponsored Ind 33
8.7 Sponsor Protocol Deviation Reporting 34
9 CLINICAL MONITORING PLAN 34
10 STATISTICAL CONSIDERATIONS 35
10.1 Primary Endpoint 35
10.2 Secondary Endpoints 35
10.3 Exploratory Endpoints 35
10.4 Sample Size Determination 36
10.5 Analysis of the Primary Endpoint 36
10.6 Analysis of the Secondary Endpoints 36
10.7 Analysis of Exploratory endpoints 36
10.8 Interim Futility Analysis and Early Stopping Rule 37
11 COLLABORATIVE AGREEMENTS 37

11.1 Transfers associated with correlative studies conducted under an approved protocol 37
11.2 Multi-Institutional Guidelines 37
11.2.1 Sites Reviewed by NIH Intramural IRB 37
12 HUMAN SUBJECTS PROTECTIONS 37
12.1 Rationale For Subject Selection 37
12.2 Participation of Children 37
12.3 Evaluation of Benefits and Risks/Discomforts 38
12.3.1 Risk/Benefit Assessment 38
12.4 Consent Process and Documentation 39
13 REGULATORY AND OPERATIONAL CONSIDERATIONS 40
13.1 Study Discontinuation and Closure 40
13.2 Quality Assurance and Quality Control 40
13.3 Conflict of Interest Policy 40
13.4 Confidentiality and Privacy 41
14 PHARMACEUTICAL INFORMATION/COMMERCIAL IMAGING 42
14.1 ^{18}F-DCFPyL (IND: 133631) 42
14.1.1 Source/Acquisition and Accountability 42
14.1.2 Toxicity 42
14.1.3 Formulation and Preparation 42
14.1.4 Stability and Storage 42
14.1.5 Administration Procedures 43
14.2 ^{18}F-FDG (IND: 133631) 43
14.2.1 Source/Acquisition and Accountability 43
14.2.2 Administration Procedures 43
14.3 Commercial Imaging 43
15 REFERENCES 44
16 APPENDICES 47
16.1 Appendix A - Performance Status Criteria 47

STATEMENT OF COMPLIANCE

The trial will be carried out in accordance with International Conference on Harmonisation Good Clinical Practice (ICH GCP) and the following:

- United States (US) Code of Federal Regulations (CFR) applicable to clinical studies (45 CFR Part 46, 21 CFR Part 50, 21 CFR Part 56, 21 CFR Part 312, and/or 21 CFR Part 812)

National Institutes of Health (NIH)-funded investigators and clinical trial site staff who are responsible for the conduct, management, or oversight of NIH-funded clinical trials have completed Human Subjects Protection and ICH GCP Training.

The protocol, informed consent form(s), recruitment materials, and all participant materials will be submitted to the Institutional Review Board (IRB) for review and approval. Approval of both the protocol and the consent form must be obtained before any participant is enrolled. Any amendment to the protocol will require review and approval by the IRB before the changes are implemented to the study. In addition, all changes to the consent form will be IRB-approved; an IRB determination will be made regarding whether a new consent needs to be obtained from participants who provided consent, using a previously approved consent form.

1 INTRODUCTION

1.1 Study Objectives

1.1.1 Primary Objective

- To assess the ability of ^{18}F-DCFPyL PET/CT imaging to detect sites of hepatocellular carcinoma (HCC).

1.1.2 Secondary Objectives

- Compare the ability of ^{18}F-DCFPyL PET/CT to detect HCC with standard of care imaging (CT and/or MRI), and ^{18}F-FDG PET/CT.
- To assess the ability of ^{18}F-DCFPyL PET/CT imaging to assess treatment response to local therapy.

1.1.3 Exploratory Objectives

- Compare the distribution of ^{18}F-DCFPyL uptake with standard of care CT and/or MRI.
- Compare focal abnormal ^{18}F-DCFPyL uptake with focal abnormalities identified on ^{18}F-FDG PET/CT.
- Compare PSMA expression with ^{18}F-DCFPyL uptake.
- Compare tumor viability and metabolism determined by ^{18}F-FDG PET imaging with ^{18}F-DCFPyL uptake.
- Evaluate ^{18}F-DCFPyL uptake with 5 year overall survival and 5 year progression free survival.
- Evaluate tumor biomarkers in blood, urine and tissue samples.

1.2 Background and Rationale

1.2.1 ^{18}F-DCFPyL

Dr. Pomper's group at Johns Hopkins University developed a second-generation low molecular weight, PSMA targeted radiotracer, 2-(3-(1-carboxy-5-[(6-[^{18}F] fluoro-pyridine-3-carbonyl)-amino]-pentyl)-ureido)-pentanedioic acid (^{18}F-DCFPyL). This compound is similar to the first-generation molecule but with enhanced characteristics. They found tissue binding affinity to be more than 5 times greater than ^{18}F-DCFBC and with significantly less blood pool activity. Their preclinical studies in prostate cancer mice models revealed a maximum target to muscle background ratio of 400:1 at 120 minutes compared to ^{18}F-DCFBC's ratio of 20:1 [1]. They noted comparably favorable pharmacokinetics and dosimetry profile.

In their first-in-human study [2], nine prostate cancer participants were evaluated with this tracer to assess safety, biodistribution and radiation dosimetry with favorable results. Physiologic radiotracer activity is seen in the salivary glands, lacrimal glands, liver, spleen and intestines with excretion through the kidneys and bladder. Blood pool activity rapidly cleared. The effective dose for a 370 MBq (8 mCi) dose of ^{18}F-DCFPyL was 6.1 mGy (0.61 rem) or 0.0165 mSv/MBq. The highest radiation dose is the kidneys (0.0945 mGy/MBq) then the bladder wall

(0.0864 mGy/MBq) which can be ameliorated by continuous bladder irrigation, submandibular glands (0.0387 mGy/MBq) and liver (0.0380 mGy/MBq). Radiation dose estimates for the other organs are in **Table 1**. More recently, several publications from the Johns Hopkins group have studied the viability of using ^{18}F-DCFPyL in prostate cancer staging [3], biochemical recurrence [4] and metastatic disease [5, 6].

Table 1. Radiation dose estimates for ^{18}F-DCFPyL

Organ	Absorbed dose (mGy/MBq)
Adrenals	3.11E-02
Brain	2.19E-03
Breasts	4.57E-03
Gallbladder wall	1.44E-02
Heart wall	1.29E-02
Kidneys	9.45E-02
Lacrimal glands	3.50E-02
Lens	1.25E-03
Liver	3.80E-02
LLI wall	1.05E-02
Lungs	1.08E-02
Muscle	6.32E-03
Osteogenic cells	9.58E-03
Ovaries	8.89E-03
Pancreas	2.44E-02
Parotid glands	2.68E-02
Red marrow	1.04E-02
Skin	4.05E-03
Small intestine	9.13E-03
Spleen	1.85E-02
Stomach wall	1.16E-02
Submandibular glands	3.87E-02
Testes	1.01E-02
Thymus	5.56E-03
Thyroid	8.56E-03
ULI wall	1.67E-02
Urinary bladder wall	8.64E-02
Uterus	1.15E-02

Tumor uptake was much higher with ^{18}F-DCFPyL than ^{18}F-DCFBC and increased with time. Focal radiotracer activity could be seen in some lesions at the earliest imaging point at 5 minutes and all known lesions were visualized with highest uptake after 2 hours post-injection. ^{18}F-DCFPyL uptake identified cancer lesions in the bone, prostate, lymph nodes and soft tissue. They did note that most lesions were visualized by 1 hour post-injection; however, there were a few small lesions only visible at 2 hours. They suggested that participants with biochemical recurrence might be best imaged at the 2 hour point (**Figure 1**).

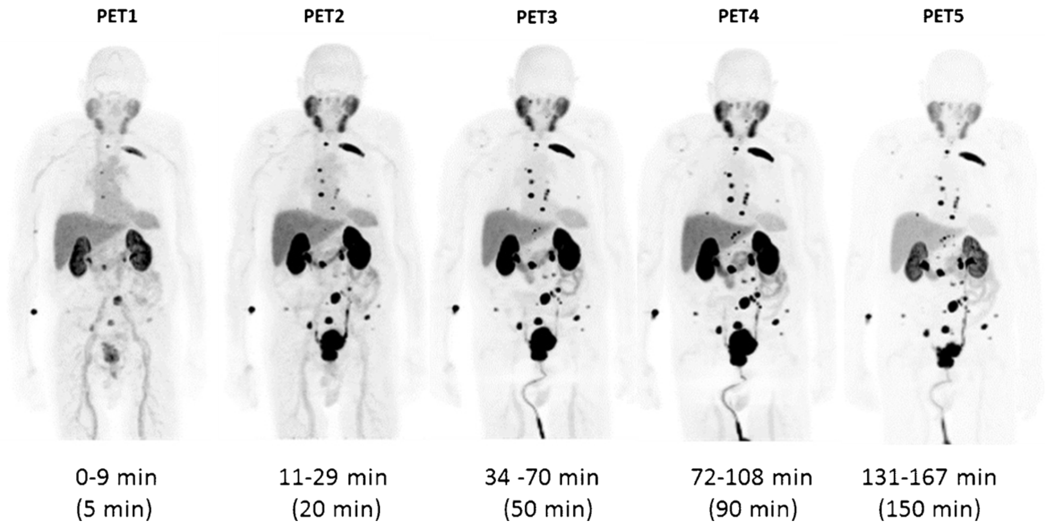

Figure 1. ^{18}F-DCFPyL PET images of a prostate cancer participant with multiple bone and lymph node disease.

1.2.2 Toxicity and Pharmacology

1.2.2.1 Toxicity in Animals

A toxicity report "14-Day Study to Determine Toxicity of ^{18}F-DCFPyL from a Single Intravenous (IV) Dose in Sprague Dawley Rats", SoBran Study Number: SB-MP-001, was performed by SoBran Inc. The following summary is taken from that report.

The purpose of this study was to evaluate the toxicity of ^{18}F-DCFPyL following a single intravenous dose in rats. This study consisted of two test article treatment groups of ten male and female Sprague-Dawley rats per group dosed with DCFPyL at 0.1 and 0.5 mg/kg. An additional group of ten males and ten females received the vehicle, 5% Dextrose, and served as the control. All rats received a dose volume of 5 mL/kg. The rats were dosed intravenously once on Study Day 1. Five male and five female rats from each group were bled on Study Day 3 and the remaining rats were bled on Study Day 15. All animals were euthanized and necropsied following blood collection. Parameters evaluated for test article effect included survival, clinical observations, body weight, body weight gain, clinical pathology, gross pathology, organ weights, and microscopic pathology.

All rats survived to the scheduled termination and remained bright, alert and responsive during the study. No abnormal findings were indicated during cage-side or hands-on observations. One female rat treated with the vehicle control and one female rat treated with 0.1 mg/kg DCFPyL lost weight between Days 8 and 15. All rats treated with 5% Dextrose or DCFPyL gained weight during the course of the study.

There were no treatment related changes seen in the hematology, coagulation, or clinical chemistry data. Organ weights showed some variance but microscopic findings in the Day 3 and Day 15 rats were considered incidental and not directly related to the test article.

Under the conditions of this study, there were no treatment related findings in Sprague Dawley rats three or fifteen days after a single intravenous dose of ^{18}F-DCFPyL at 0.1 mg/kg and 0.5 mg/kg.

Thus, similar toxicity was observed for the second-generation compound DCFPyL and was awarded a physician sponsored FDA exploratory IND based on this acceptable pre-clinical data (IND#121,064) for ^{18}F-DCFPyL as a PET radiopharmaceutical, which is held by Dr. Martin Pomper.

^{18}F-DCFPyL is currently approved by the FDA as a radioactive diagnostic agent indicated for positron emission tomography (PET) of prostate-specific membrane antigen (PSMA) positive lesions in men with prostate cancer: with suspected metastasis who are candidates for initial definitive therapy or with suspected recurrence based on elevated serum prostate specific antigen (PSA) level.

1.2.2.2 Toxicity in Humans

Szabo et al [7] first reported the use of ^{18}F-DCFPyL in five participants with metastatic prostate cancer and none experienced any severe adverse events. In recent trials at NCI using ^{18}F-DCFPyL, Mena et al. [8] and Gaur et al. [9] reported no adverse events in prospective studies including 90 participants with biochemical recurrence prostate cancer, and 26 participants with localized prostate cancer, respectively. Another group has proved ^{18}F-DCFPyL to be safe in a larger population of 130 subjects with biochemical recurrence prostate cancer [10]. Three grade I NCI CTCAE adverse events were reported. One participant reported a mild headache and nosebleed (two adverse events) that resolved without treatment and were deemed unlikely to be attributed to the radiotracer. The third event was a decreased platelet count found on routine post-imaging follow-up labs that was attributed to the participant starting treatment for prostate cancer. No heart rate or blood pressure events occurred that were related to the radiotracer.

1.2.3 Safety

The effective radiation dose to participants with ^{18}F-DCFPyL was reported as 0.0165 mSv/MBq by Szabo et al [7]. Doses to most radiosensitive organs were much lower than with the first-generation, ^{18}F-DCFBC. They compared dosing to other similar agents, such as ^{68}Ga DOTATATE, which is two times higher in effective radiation dose and ^{124}I MIP, which is at least one order of magnitude higher in all measured organs, including radiosensitive organs and the whole body.

1.3 STUDY RATIONALE

1.3.1 PSMA Expression in Human Cancers

Prostate specific membrane antigen (PSMA) has been extensively studied in prostate cancers. PSMA is overexpressed in high-grade tumors, and increases when de-differentiation, metastatic or hormone-refractory disease occur, making PSMA a prognostic factor for disease outcome [11-13]. However, PSMA is not entirely prostate-specific and it is expressed in several other neoplasms, as well as tumor-associated vasculature [14, 15]. Importantly, PSMA-expression of normal endothelium is not observed.

Several PET radiotracers, including small-molecule inhibitors (known as ligands) targeting PSMA receptor have been developed, such as DCFPyL to facilitate the diagnosis of prostate cancer in humans [2, 5, 16].

1.3.2 Hepatocellular Carcinoma (HCC)

Hepatocellular carcinoma (HCC) accounts for about 80% of all primary liver cancer. It is the fifth most common cancer world-wide and the third most common cause of cancer-related mortality [17]. Current guidelines of the American Association for the Study of Liver Disease and the European Association for the Study of the Liver recommend HCC surveillance with abdominal ultrasound every 6 months in participants at high risk of developing the disease. Participants diagnosed in early stages of HCC are eligible for potentially curative therapy. Therefore, early diagnosis and accurate staging are critical for a favorable prognosis. Curative-intent therapies for HCC include surgical resection, liver transplantation, microwave or radiofrequency ablation in case of small tumors [18].

Imaging of HCC at diagnosis is challenging, and assessment of HCC recurrence after treatment is also difficult because it can be confounded by coagulative necrosis, hematomas, abscesses, and fluid or bile collections [19]. Standard of care imaging modalities used in participants with HCC are ultrasound (with or without contrast) and multiphasic CT or MRI. Each method has its shortcomings, the major one being the lack of correlation between radiologic appearance and biologic activity. The imaging of liver lesions in cirrhotic participants is complex and challenging. The cirrhotic liver acquires a nodular architecture with altered vascularity, making difficult the differentiation of regenerative nodules from early HCC and metastases from other primary tumors. The challenge is even greater in participants who undergo ablative therapies for known HCC lesions.

Hence, there is a need for an imaging modality that can be used to localize HCC tumors more accurately. Functional imaging with PET has so far not played a major role in the diagnosis or surveillance of HCC, and the most commonly used PET tracer, FDG, is not routinely used since only a minority of FDG is taken up by HCC tumors [20], with suboptimal sensitivity (<50%) for detection of HCC tumors, being inferior to CT [21].

1.3.3 Hepatocellular Carcinoma and PSMA

It has been shown that PSMA can be expressed not only on prostate cancer cells, but also on cell lines of other malignancies, as well as tumor endothelium. A recent publication reported that nearly 95% of hepatocellular cancers stained positive for PSMA in the tumor vasculature. Zhu et al. [22] suggested that the process of endothelial cell recruitment to HCC occurs early and throughout the process of hepatic tumorigenesis, making an endothelial cell tracer an ideal marker to detect early disease. In a recent study assessing 100 HCC tissue samples using immunohistochemical staining, Jiao and collaborators [23] reported PSMA expression in more than 50% of tumor-associated vasculature in 26% of tissues, and PSMA expression in less than 50% of vasculature in 48% of tissues. Furthermore, high vascular PSMA expression was also associated with poor prognosis in participants with HCC, and could be used as an independent prognostic marker for HCC [23]. Tolkach et al. [24] found that the majority of hepatocellular carcinomas (89.9%) show high levels of PSMA expression on tumor neovasculature and on canalicular membrane of the tumor cells (4.1% of tumors) [24]. To date, most of the reported findings with PSMA-targeted PET radiotracers in HCC have been in few case reports [25] and small case series [26]. Recently, ^{68}Ga-PSMA PET imaging showed encouraging preliminary results for imaging HCC in a small cohort of 7 participants. ^{68}Ga-PSMA PET-CT showed superiority over ^{18}F-FDG PET-CT in imaging participants with HCC. All but a single tumor lesion were associated with ^{68}Ga-PSMA uptake higher than that of the surrounding liver

parenchyma (**Figure 2** and **Figure 3**), with a mean uptake 3.6 times higher than that of the background liver. The increased ^{68}Ga-PSMA uptake in HCC was corroborated by the results of immunohistochemistry analysis showing PSMA staining of the endothelial cells lining of vessels that are penetrated by tumor. Furthermore, ^{68}Ga-PSMA PET appeared to differentiate necrotic from viable tumor (**Figure 3**), which could dramatically improve detection of HCC recurrence following local ablative therapies [26]. In another recent study, Kuyumcu et al. [27] was able to visualize advanced HCC using ^{68}Ga-PSMA-PET/CT in 16 out of 19 cases with high tumor-to-background ratio. Interestingly, ^{68}Ga-PSMA standard uptake value (SUVmax) correlated with participant overall survival [27].

Few direct comparisons between ^{18}F-DCFPyL and ^{68}Ga-PSMA-PET have been reported in prostate cancer population and only in one manuscript. Dietlein et al. [28] reported comparable biodistributions for both tracers, but slightly higher sensitivity for ^{18}F-DCFPyL compared to ^{68}Ga-PSMA-11 (88% vs 66%) in participants with biochemical recurrence of prostate cancer.

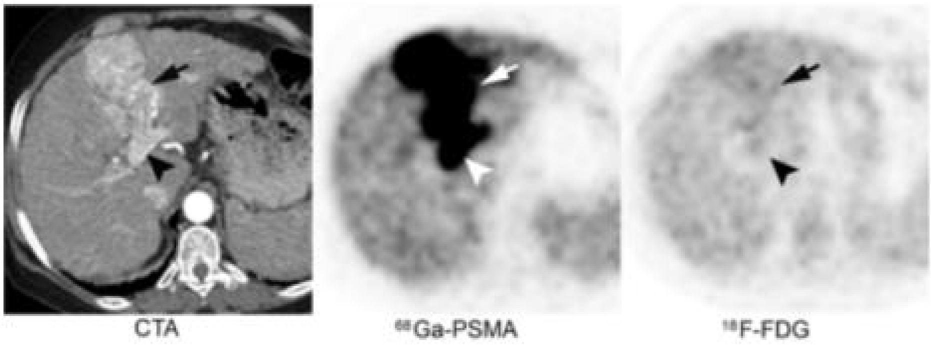

Figure 2. Enhancing HCC. Increased uptake of 68Ga-PSMA was seen within the tumor at the left liver lobe (arrows) as well as in tumor thrombus in the left portal vein (arrowheads). These lesions showed no significant FDG avidity.

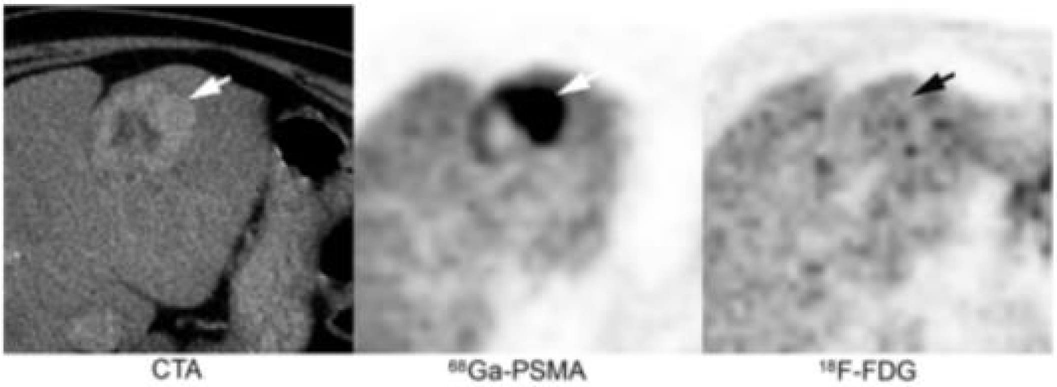

Figure 3. HCC lesions with mosaic contrast enhancement on CT angiography. Increased ^{68}Ga-PSMA uptake was identified only in the enhancing area (arrows). Lesions were non-FDG-avid.

1.3.4 ^{18}F-DCFPyL in HCC

We propose to expand clinical work using ^{18}F-DCFPyL PET imaging in prostate cancer participants, and evaluate its usefulness in detecting sites of hepatocellular carcinoma. ^{18}F-DCFPyL distribution and tumor uptake will be compared with the results of CT, and /or MRI.

This may provide data to support a future trial using ^{18}F-DCFPyl PET/CT, define its role in the diagnosis and surveillance of HCC, especially following treatment. This study should offer insights into how and when ^{18}F-DCFPyL could be used in the clinical setting to direct proper management.

2 ELIGIBILITY ASSESSMENT AND ENROLLMENT

2.1 ELIGIBILITY CRITERIA

2.1.1 Inclusion Criteria

2.1.1.1 High radiological suspicion of hepatocellular carcinoma (LR4 or LR5 based on the most current version of LI-RADS) with at least one measurable lesion on standard imaging modality (CT and/or MRI).

2.1.1.2 Eligible for local therapies (included but not limited to surgical resection, stereotactic radiation therapy, transarterial chem/radio/bland embolization, microwave ablation, radiofrequency ablation).

2.1.1.3 Ability to take oral medication and be willing to adhere to the study intervention regimen.

2.1.1.4 Age $\geq$18 years.

2.1.1.5 ECOG performance status $\leq$2 (see **Appendix A**).

2.1.1.6 Known human immunodeficiency virus (HIV)-infected individuals must be on effective anti-retroviral therapy with undetectable viral load within 6 months.

2.1.1.7 Known chronic hepatitis B virus (HBV) infected individuals, must be on suppressive therapy with undetectable viral load.

2.1.1.8 Individuals with a history of hepatitis C virus (HCV) infection must have been treated and cured.

2.1.1.9 The effects of ^{18}F-DCFPyL (study drug) on the developing human fetus are unknown. For this reason and because this agent as well as other therapeutic agents used in this trial are known to be teratogenic, women of child-bearing potential and men must agree to use adequate contraception (hormonal or barrier method of birth control; abstinence) prior to study entry and for 2 months after each study PET/CT imaging. Should a woman become pregnant or suspect she is pregnant while she or her partner is participating in this study, she should inform her treating physician immediately.

2.1.1.10 Ability of subject to understand and the willingness to sign a written informed consent document.

2.1.2 Exclusion Criteria

2.1.2.1 History of allergic reactions attributed to compounds of similar chemical or biologic composition to ^{18}F-DCFPyL or other agents used in study.

2.1.2.2 Uncontrolled intercurrent illness including, but not limited to, ongoing or active infection, symptomatic congestive heart failure, unstable angina pectoris, cardiac arrhythmia, or psychiatric illness/social situations that would limit compliance with study requirements.

2.1.2.3 Subjects with severe claustrophobia unresponsive to oral anxiolytics.

2.1.2.4 Other medical conditions deemed by the principal investigator (or associates) to make the subject unsafe/ineligible for protocol procedures.

2.1.2.5 Subjects weighing > 350 lbs (weight limit for scanner table), or unable to fit within the imaging gantry.

2.1.2.6 Serum creatinine > 2 times the upper limit of normal.

2.1.2.7 Pregnant women are excluded from this study because ^{18}F-DCFPyL is an agent with the potential for teratogenic or abortifacient effects. as well as other agents used in this trial are known to be teratogenic.

2.2 RECRUITMENT STRATEGIES

This protocol may be abstracted into a plain language announcement posted on NIH websites and on NIH social media platforms. Participants will also be identified via internal referrals from each participating site institution, and outside referrals.

2.3 SCREENING EVALUATION

2.3.1 Screening activities performed prior to obtaining informed consent

Minimal risk activities that may be performed before the subject has signed a consent include the following:

- Email, written, in person or telephone communications with prospective subjects
- Review of existing medical records to include H&P, laboratory studies, etc.
- Review of existing MRI, x-ray, or CT images
- Review of existing photographs or videos
- Review of existing pathology specimens/reports from a specimen obtained for diagnostic purposes

2.3.2 Screening activities performed after a consent for screening has been signed

The following activities will be performed only after the subject has signed the study consent OR the consent for study 01-C-0129 (for NIH only provided the procedure is permitted on that study) on which screening activities may also be performed. Assessments performed at outside facilities or on another NIH protocol within the timeframes below may also be used to determine eligibility once a participant has signed the consent.

Within 30 days prior to administration of ^{18}F-DCFPyL, unless otherwise specified:

- CT and/or MRI (outside imaging studies can be used; however, the images must be made available in DICOM format for review by MIB staff) (within 2 months prior to administration of ^{18}F-DCFPyL).
- Medical history, concomitant medication review and physical examination (including height, weight, vital signs, and ECOG performance status).
- Hematological profile: CBC with differential and platelet count
- Acute care profile: sodium, potassium, chloride, total CO_2, creatinine, glucose, urea nitrogen, eGFR
- Serum or urine pregnancy test for female participants of childbearing age (in the absence of prior hysterectomy).

2.4 Screen Failures

Screen failures are defined as participants who consent to participate in the clinical trial but are not subsequently assigned to the study intervention or entered in the study. A minimal set of screen failure information is required to ensure transparent reporting of screen failure participants, to meet the Consolidated Standards of Reporting Trials (CONSORT) publishing requirements and to respond to queries from regulatory authorities. Minimal information includes demography, screen failure details, eligibility criteria, and any serious adverse event (SAE).

Individuals who do not meet the criteria for participation in this trial (screen failure) because prior biopsy did not confirm HCC may be rescreened.

2.5 Intervention Assignment Procedures

Cohorts

**Number**	**Name**	**Description**
1	HCC	Participant with radiographically confirmed hepatocellular cancer.

Arms

**Number**	**Name**	**Description**
1	Baseline and Post-treatment Imaging	^{18}F-DCFPyL PET/CT imaging, CT and/or MRI and standard of care local treatment.

Arm assignment

Participants in cohort 1 will directly be assigned to arm 1.

3 STUDY IMPLEMENTATION

3.1 STUDY DESIGN

This is an open-label multi-center study recruiting participants with suspected hepatocellular carcinoma (with at least one detectable liver lesion on standard of care CT and/or MRI) who are eligible to undergo local treatment for HCC (e.g., surgical resection, radiofrequency ablation, microwave ablation, transarterial embolization (TAE), stereotactic body radiotherapy (SBRT)). Participants will be consented and enrolled through the NIH Clinical Center.

All participants will undergo a baseline ^{18}F-DCFPyL PET/CT. Within approximately 2 weeks of each ^{18}F-DCFPyL PET/CT scan, participants will also be scanned with a ^{18}F-FDG PET/CT imaging. ^{18}F-FDG PET/CT may be performed within two weeks before or after each ^{18}F-DCFPyL PET/CT scan. Each PET/CT imaging must be approximately a day apart. The order obtained for ^{18}F-DCFPyL PET/CT and ^{18}F-FDG PET/CT does not matter.

A CT and/or MRI will be performed within 2 months of the ^{18}F-DCFPyL PET/CT scan. Clinical scans performed in this time frame by local providers are also acceptable.

Participants will undergo a biopsy prior to/concurrent with local therapy. The procedure may be performed at the NIH Clinical Center or at the participating site (Washington DC Veteran's Affairs Medical Center). The treatment procedure will be scheduled as clinically indicated and samples will be collected prior to therapy (or during surgical resection).

An additional ^{18}F-DCFPyL PET/CT imaging will be performed during the first routine post local therapy follow-up for participants with a positive baseline ^{18}F-DCFPyL-PET/CT (i.e. the presence of DCFPyL-avid tumor/s) and with a biopsy confirming HCC diagnosis. Subjects with negative tumor uptake at the baseline ^{18}F-DCFPyL-PET/CT and with a biopsy confirming HCC, will not have study-related imaging post-treatment and will remain in follow-up. Subjects with biopsies negative for HCC will be taken off protocol.

Participants with a positive HCC biopsy will be followed by clinical chart review, phone-call, email follow-up or any other NIH approved remote platform for tumor markers and radiologic evidence of recurrence over a 5-year period from the last ^{18}F-DCFPyL PET/CT imaging. A set of imaging (^{18}F-DCFPyL PET/CT, ^{18}F-FDG PET/CT, CT/MRI) may be performed and research samples may be collected at the time of tumor recurrence per PI discretion. Should participants undergo another treatment during the follow up period, a biopsy may be done per PI discretion.

For detailed study and schedule of procedures, refer to section **3.3**.

3.2 AGENT ADMINISTRATION

3.2.1 ^{18}F-DCFPyL administration

The target administered activity will be 9 mCi; dose variations will be in accordance with the Nuclear Regulatory Commission (NRC) standard dose variation (i.e. 20%) permitted for diagnostic clinical studies. Due to potential unpredictable delays and the short half-life of ^{18}F, the total dose of ^{18}F-DCFPyL administered may be reduced at the discretion of the principal investigator or their designee.

The administration site should be evaluated just before, during and after injection, to assess for extravasation and/or for the presence of signs of local irritation.

Because there is an unknown but potential risk for adverse events in nursing infants secondary to exposure of the mother to 18F, breastfeeding should be discontinued for 12 hours after administration of either ^{18}F-DCFPyL or ^{18}F-FDG.

3.2.2 ^{18}F-DCFPyL PET/CT imaging

The ^{18}F-DCFPyL PET/CT imaging will consist of the ^{18}F-DCFPyL injection, followed by a ~45 min dynamic CT imaging of a single bed position (including the liver lesion), and a static whole-body PET/CT imaging (top of head to mid-thighs) performed at 1 hour (+/- 10 minutes) post ^{18}F-DCFPyL injection. Only a single injection of ^{18}F-DCFPyL is required. The initial 45 minutes dynamic regional scan will be used to determine the kinetics of ^{18}F-DCFPyL within the tumor as compared with normal liver and other background.

Participants will be encouraged to hydrate and urinate frequently after administration of the drug.

Summary of scanning procedure:

1. IV placement
2. Administration of ^{18}F-DCFPyL; begin AE monitoring
3. Dynamic PET/CT imaging of a single field-of-view (including the liver lesion) will be performed (~ 45 minutes)
4. Subject asked to void
5. 1 hour (+/- 10 minutes) following ^{18}F-DCFPyL injection, a static whole body PET/CT imaging will be performed (~30-45 minutes in duration)
6. Follow-up AE query ~1-3 days post-injection

3.2.3 ^{18}F-FDG PET/CT imaging

The ^{18}F-FDG PET/CT imaging will consist of an ^{18}F-FDG injection and PET/CT imaging performed approximately 1 hour post ^{18}F-FDG injection. A corresponding low dose CT scan for attenuation correction and co-registration purposes will be performed prior to the PET image.

3.3 STUDY CALENDAR

**Procedures**	**Screening**[1]	**Baseline** [1] **Study Visit 1**	**Study Visit 2** [10]	**Follow-up** [11]	**Disease progression**
Informed consent	X				
**Medical Assessment**					
Demographics	X				
Medical history	X				
Concomitant medication review	X				
**Physical Assessment**					
ECOG	X				
Physical exam (including height and weight)	X				
Vital signs	X	X **2**	X **2**		
**Laboratory Assessment**					
Hematology (CBC with diff and platelet)	X		X		
Serum chemistry/Acute Care Panel **3**	X		X		
Serum or urine pregnancy test **4**	X		X		X
**Study Intervention**					
CT and/or MRI	X **5**		X **5**		X **12**
^{18}F-DCFPyL PET/CT imaging **6**		X	X		X **12**
^{18}F-FDG PET/CT imaging **7**		X	X		X **12**
**Adverse Event Monitoring**					
Injection site monitoring **8**		X	X		X
Adverse event monitoring/Query **8**		X	X		X

**Procedures**	**Screening**[1]	**Baseline** [1] **Study Visit 1**	**Study Visit 2** [10]	**Follow-up** [11]	**Disease progression**
**Correlative studies**					
Tumor marker tests 9		X	X		X
Exome germline control blood collection		X			
Tumor Biopsy		Concurrent with standard of care therapy (pre-treatment or during surgical resection)			
Blood and urine for EVP analysis		X	X	X	X
**Follow-up evaluations**					
Review of clinical chart, phone call or e-mail (or any NIH approved platform)				X	
Collection/review of imaging scans				X	

1. Performed within 30 days prior to administration of ^{18}F-DCFPyL unless otherwise indicated. Screening procedures such as medical assessment may be performed remotely via any NIH approved platforms.
2. Vital signs will be taken prior to injection of ^{18}F-DCFPyL, and following completion of the final PET/CT scan (+ 15 minutes).
3. Acute care panel: sodium, potassium, chloride, total CO_2, creatinine, glucose, urea nitrogen, eGFR
4. For female participants of childbearing age (in the absence of prior hysterectomy). Pregnancy tests may be performed as clinically indicated prior to each scan.
5. CT (Chest/Abdomen/Pelvis) and/or MRI (Abdomen) will be performed within 2 months of each ^{18}F-DCFPyL PET/CT. Imaging may be performed at certified outside facility and provided to study team.
6. Subjects will undergo ^{18}F-DCFPyL injection and a dynamic PET/CT. Approximately 1 hour (+/- 10 minutes) post ^{18}F-DCFPyL injection, a static PET/CT imaging performed. Refer to section **3.2.2** for additional information regarding the scanning procedure.
7. ^{18}F-FDG PET/CT will be performed within approximately 2 weeks before or after each ^{18}F-DCFPyL scan. Each PET/CT imaging must be approximately a day apart. The order obtained for ^{18}F-DCFPyL PET/CT and ^{18}F-FDG PET/CT does not matter.
8. Event monitoring will be done at the time of injection, and 1 hour post injection. All subjects will be contacted by phone at ~1-3 business days post-injection and will be asked non-leading questions regarding symptoms. At the investigator's discretion, subjects

with safety concerns noted during the post injection period may remain at the site or be asked to return to the site to undergo further safety assessments at the 1-3 business day follow-up time point.

9. Tumor markers include Alpha-fetoprotein, carcinoembryonic antigen, carbohydrate antigen 19.9 (CA19.9) and liver function tests (Alkaline Phosphatase, ALT, AST, Total Bilirubin, Direct Bilirubin). Refer to section **5.1** for more details.
10. Only participants with a positive baseline ^{18}F-DCFPyL-PET/CT scan (i.e. with the presence of DCFPyL-avid tumor/s) and a biopsy confirming HCC diagnosis will undergo visit 2 for a second ^{18}F-DCFPyL PET/CT (during routine treatment follow up, typically within 4-8 weeks).
11. Follow-up will be performed every 3 months after the last ^{18}F-DCFPyL scan (or after therapy for participants with negative baseline ^{18}F-DCFPyL PET/CT and biopsy confirming HCC) for 2 years, and yearly afterwards for an additional 3 years.
12. If tumor recurrence occurs during the follow-up period, an ^{18}F-DCFPyL PET/CT, ^{18}F-FDG PET/CT, CT/MRI and biopsy may be performed any time after recurrence at PI discretion. Biopsy would be performed concurrently with treatment if the latter is performed.

3.4 SURGICAL GUIDELINES

Participants may undergo medically indicated surgical procedures for diagnostic and/or therapeutic intervention on this protocol as per standard of care for HCC. No surgery will be performed solely for the purpose of obtaining research specimens.

3.5 COST AND COMPENSATION

3.5.1 Costs

Subjects' costs will be based on local guidelines as described in the site specific consent.

3.5.2 Compensation

Participants will not be compensated on this study.

3.6 CRITERIA FOR REMOVAL FROM PROTOCOL INTERVENTION AND OFF STUDY CRITERIA

Prior to removal from study, effort must be made to have all subjects complete a safety visit by phone approximately ~1-3 business days following the last dose of study intervention.

3.6.1 Criteria for removal from protocol intervention

- PI discretion
- A serious or intolerable event related to the study agent occurs
- Participant requests to be withdrawn from active intervention
- ^{18}F-DCFPyL is no longer available

3.6.2 Off-Study Criteria

- Participants with biopsy that does not confirm HCC diagnosis
- Participant requests to be withdrawn from the study
- Participant becomes pregnant
- PI discretion
- Subject becomes decisionally impaired
- Lost to follow up
- Death
- PI decision to close the study
- Completion of follow up period
- Screen failure

3.6.3 Lost to Follow-up

A participant will be considered lost to follow-up if he or she fails to return for 2 scheduled visits and is unable to be contacted by the study site staff on at least two (2) separate occasions.

The following actions must be taken if a participant fails to return to the clinic for a required study visit:

- The site will attempt to contact the participant and reschedule the missed visit and counsel the participant on the importance of maintaining the assigned visit schedule and ascertain if the participant wishes to and/or should continue in the study.
- Before a participant is deemed lost to follow-up, the investigator or designee will make every effort to regain contact with the participant (where possible, 3 telephone calls and, if necessary, an IRB approved certified letter to the participant's last known mailing address or local equivalent methods). These contact attempts should be documented in the participant's medical record or study file.
- Should the participant continue to be unreachable, he or she will be considered to have withdrawn from the study with a primary reason of lost to follow-up.

4 CONCOMITANT MEDICATIONS/MEASURES

In the event that a subject has a reaction (allergic) to the radiotracer, all appropriate medical measures will be taken immediately. In rare instances, this may entail admission to the hospital for observation.

Participants are permitted to undergo systemic therapy for their disease during the course of the study. However, an attempt should be made to maintain the same systemic therapy during the two sets of scans to avoid confounding.

Participants may also receive standard of care therapy on this study.

5 CORRELATIVE STUDIES FOR RESEARCH

5.1 Biospecimen Collection

Table 2. Summary of Collection of Correlative Samples

Test/assay	Volume (approx)	Type of tube [a]	Collection timepoint	Location of specimen	Sample data system used
**Tissue samples**					
IHC	4 cores (18G)	NA	Concurrently with standard of care treatment	Laboratory of Pathology (LP)/COMPASS<br>Blood Processing Core (Figg lab)<br>CCR Sequencing Facility<br>Xin Wang laboratory<br>Participating sites should process and ship samples to Figg lab per section 5.2.	Labmatrix
PSMA staining					
Next Generation Sequencing					
**Blood samples**					
Alpha-fetoprotein	3.5 mL	1 SST	Within 30 days of each ^{18}F-DCFPyL PET/CT imaging	Participating site Clinical Center laboratory<br>or<br>Outside Clinical facility	C3D
Carcinoembryonic antigen					
Carbohydrate antigen 19.9					
Liver function tests (Alkaline Phosphatase, ALT, AST, Total Bilirubin, Direct Bilirubin)	3.5 mL	1 Green Lithium heparin			

Test/assay	Volume (approx)	Type of tube [a]	Collection timepoint	Location of specimen	Sample data system used
Germline Mutation Control	3 mL	1 Sodium Citrate tube	Baseline	Laboratory of Pathology/COMPASS	Labmatrix
EVP analysis	3.5 mL	2 SST tubes	Baseline, Study Visit 2 Follow-up Progression	Figg lab	Labmatrix
	10 mL	2 Streck DNA BCT	Baseline Study Visit 2 Follow-up Progression	Figg lab	Labmatrix
	20 mL	2 Streck complete (RNA, EV) BCT	Baseline Study Visit 2 Follow-up Progression	Figg lab	Labmatrix
**Urine Sample**					
EVP analysis	50mL	Urine cup	Baseline Study Visit 2 Follow-up Progression	Figg lab	Labmatrix

a. Please note that tubes and media may be substituted based on availability with the permission of the PI or laboratory investigator.

5.1.1 IHC and PSMA Staining on Tissue Samples

Specimen (1 core) will undergo routine formalin fixation and paraffin embedding, immunohistochemistry, tumor diagnosis, and molecular evaluation in the NCI Laboratory of Pathology. PSMA staining will also be conducted. The specimen results will be correlated with ^{18}F-DCFPyL PET/CT and standard CT and/or MRI imaging results.

5.1.2 HCC Blood Biomarker Studies

Alpha-fetoprotein, carcinoembryonic antigen, carbohydrate antigen 19.9 (CA19.9) and liver function tests (Alkaline Phosphatase, ALT, AST, Total Bilirubin, Direct Bilirubin) will be performed (may done by an outside facility) within 30 days of each ^{18}F-DCFPyL PET/CT imaging.

5.1.3 Analysis of Extracellular Vesicles and Particles (EVPs)

Blood and urine samples will be used in the Jones Lab (stored in Figg lab) for analysis of extracellular vesicles and particles (EVPs). Specifically, we are most interested in measuring the concentration and composition of the EVPs that are PSMA+ across the samples, as related to PSMA detection on imaging and in response to treatment. We have identified robust methods for detecting and isolating PSMA+ EVPs, and then evaluating co-expressed proteins and nucleic acids associated with those EVPs. EVPs in general will be processed and evaluated according to our published protocols (nano.ccr.cancer.gov), with PSMA+ EVPs enriched by immunoaffinity from cell-free blood and urine samples. Our hypothesis is that the molecular cargo and composition of the PSMA+ EVPs in liquid biopsies (ie, accessible in blood and urine samples) may correlate with 1) imaging extent of PSMA detection, 2) additional histopathologic features identified in the targeted biopsies in the study, and 3) the biological / clinical course of PSMA+ foci, followed over time, including, but not limited to, local control and time to progression.

The following analysis will be performed:

1. Isolation of EVPs will be performed with size exclusion and affinity chromatography.
2. Enumeration and characterization of EVPs and their composition will be performed with nanoparticle tracking analysis, resistive pulse sensing, and flow cytometry to determine whether volume of PSMA+ tissue on imaging correlates with concentration of PSMA+ EVPs in blood and/or urine. Routine laboratory assays for protein/DNA/RNA quantification will also be performed.

Where sufficient cell free biofluid is available, NGS DNA methylomics will be compared between pooled PSMA+ (or other surface marker positive) EVP vs PSMA- (marker negative) vs general biofluid isolates to identify distinctive EVP subset cargo signatures that correlate with disease extent, progression, or other clinical / histopathologic correlates.

5.1.4 Next Generation Sequencing from Tissue and Blood Samples

In order to address the exploratory goal of characterizing predictive biomarkers of response, including but not limited to genomic, transcriptomic (RNA Seq), and/or epigenomic assessments of tumor tissue may be performed. Tissue samples will be sent to the Wang laboratory, Figg laboratory, Laboratory of Pathology (via Dr. Aldape's COMPASS program), and CCR Sequencing Facility at Frederick National Laboratory for Cancer Research to conduct this

analysis. A blood sample will be sent to the Laboratory of Pathology for germline mutation control.

5.2 Instructions for Participating Sites to Ship Tumor Samples

Instructions for collection and shipping of samples will be provided directly to site.

5.3 Sample Storage, Tracking and Disposition

All specimens obtained in the protocol are used as defined in the protocol. Any specimens that are remaining at the completion of the protocol will be stored in the conditions described below. The study will remain open so long as sample or data analysis continues. Samples from consenting subjects will be stored until they are no longer of scientific value or if a subject withdraws consent for their continued use, at which time they will be destroyed.

If the participant withdraws consent the participants data will be excluded from future distributions, but data that have already been distributed for approved research use will not be able to be retrieved.

The PI will record any loss or unanticipated destruction of samples as a deviation. Reporting will be per the requirements of section **7.2**. Participating sites must inform coordinating site PI of any loss or unanticipated destruction of samples per section **7.3**.

Samples will be barcoded and stored under conditions specified by research laboratory. Sample barcodes are linked to participant demographics and limited clinical information. Access to stored clinical samples is restricted. Samples will be stored until requested by a researcher named on the protocol. All requests are monitored and tracked in an NIH approved sample storage and tracking system.

5.4 Samples for Genetic/Genomic Analysis

5.4.1 Description of the scope of genetic/genomic analysis

Refer to section **5.1.4** of the protocol.

5.4.2 Management of Results

Subjects will be contacted if a clinically actionable gene variant is discovered. Clinically actionable findings for the purpose of this study are defined as disorders appearing in the American College of Medical Genetics and Genomics recommendations for the return of incidental findings that is current at the time of primary analysis. (A list of current guidelines is maintained on the CCR intranet: https://ccrod.cancer.gov/confluence/display/CCRCRO/Incidental+Findings+Lists. Subjects will be contacted at that time with a request to provide a blood sample to be sent to a CLIA certified laboratory.

For samples undergoing analysis through the NCI COMPASS, results will be reported per CCR SOP ADGC-5, Tumor/Normal Whole Exome Sequencing: Consenting, Ordering, and Obtaining Results found here.

5.4.3 Genetic Counseling

Genetic counseling will be performed per local institutional guidelines if any incidental findings are returned.

6 DATA COLLECTION AND EVALUATION

6.1 DATA COLLECTION

The PI at each site will be responsible for overseeing entry of data into a 21 CFR Part 11 compliant data capture system provided by the NCI CCR and ensuring data accuracy, consistency, and timeliness. Data quality assurance will be performed per sections **7.4** and **9**. Data should be received by the coordinating institution at least quarterly.

All adverse events, including clinically significant abnormal findings on laboratory evaluations, regardless of severity, will be followed until return to baseline or stabilization of event.

Document AEs from the first study intervention through 3 days after the agent was last administered. Beyond the 3 days after the last intervention and through the remaining follow-up, only adverse events which are serious need to be recorded.

An abnormal laboratory value will be recorded in the database as an AE **only** if the laboratory abnormality is characterized by any of the following:

- Results in discontinuation from the study
- Is associated with clinical signs or symptoms
- Requires treatment or any other therapeutic intervention
- Is associated with death or another serious adverse event, including hospitalization.
- Is judged by the Investigator to be of significant clinical impact
- If any abnormal laboratory result is considered clinically significant, the investigator will provide details about the action taken with respect to the test drug and about the participant's outcome.

End of study procedures: Data will be stored according to HHS, FDA regulations, and NIH Intramural Records Retention Schedule as applicable.

Loss or destruction of data: Should we become aware that a major breach in our plan to protect subject confidentiality and trial data has occurred, this will be reported expeditiously per requirements in section **7.2**.

6.2 DATA SHARING PLANS

6.2.1 Human Data Sharing Plan

What data will be shared?

I will share human data generated in this research for future research as follows:

- Coded, linked data in an NIH-funded or approved public repository.
- Coded, linked data in BTRIS (automatic for activities in the Clinical Center)
- Identified or coded, linked data with approved outside collaborators under appropriate agreements.

How and where will the data be shared?

- Data will be shared through:

- An NIH-funded or approved public repository. Insert name or names: Clinicaltrials.gov; dbGap.
- BTRIS (automatic for activities in the Clinical Center)
- Approved outside collaborators under appropriate individual agreements.
- Publication and/or public presentations.

When will the data be shared?

- Before publication.
- At the time of publication or shortly thereafter.

6.2.2 Genomic Data Sharing Plan

This study will comply with the NIH Genomic Data Sharing Policy, which applies to all NIH-funded research that generates large-scale human or non-human genomic data, as well as the use of these data for subsequent research. Large-scale data include genome-wide association studies (GWAS), single nucleotide polymorphisms (SNP) arrays, and genome sequence, transcriptomic, epigenomic, and gene expression data.

Therefore, unlinked genomic data will be deposited in public genomic databases such as dbGaP in compliance with the NIH Genomic Data Sharing Policy.

6.3 TOXICITY CRITERIA

The following adverse event management guidelines are intended to ensure the safety of each participant while on the study. The descriptions and grading scales found in the revised NCI Common Terminology Criteria for Adverse Events (CTCAE) version 5.0 will be utilized for AE reporting. All appropriate treatment areas should have access to a copy of the CTCAE version 5.0. A copy of the CTCAE version 5.0 can be downloaded from the CTEP web site (http://ctep.cancer.gov/protocolDevelopment/electronic_applications/ctc.htm).

6.3.1.1 Image Analysis

Target Lesions: Images will be analyzed by MIB staff by identifying, recording and measuring measurable target lesions. A segmentation tool will be applied to generate semi-automated volume of interests (VOI) within the target lesion(s). Maximum standardized uptake value (SUV_{max}) and tumor volumes will be extracted from the generated target lesion(s) VOIs. ^{18}F-DCFPyL uptake in major organs and bone marrow will also be measured and mean SUV values will be extracted.

All other lesions (or sites of disease) including any measurable lesions should be identified as non-target lesions and should also be recorded.

Antitumor effect: To determine whether uptake of ^{18}F-DCFPyL correlates with response to therapy, target tumor(s) imaging PET parameters, including SUV_{max}, and tumor volume will be compared between responders vs non-responders. Participant response will be based on RECIST 1.1. criteria and clinical assessment.

7 NIH REPORTING REQUIREMENTS / DATA AND SAFETY MONITORING PLAN

7.1 DEFINITIONS

Please refer to definitions provided in Policy 801: Reporting Research Events found here.

7.2 OHSRP OFFICE OF COMPLIANCE AND TRAINING / IRB REPORTING

7.2.1 Expedited Reporting

Please refer to the reporting requirements in Policy 801: Reporting Research Events and Policy 802 Non-Compliance Human Subjects Research found here. Note: Only IND Safety Reports that meet the definition of an unanticipated problem will need to be reported per these policies.

7.2.2 IRB Requirements for PI Reporting at Continuing Review

Please refer to the reporting requirements in Policy 801: Reporting Research Events found here.

7.3 NCI GUIDANCE FOR REPORTING EXPEDITED ADVERSE EVENTS FOR MULTI-CENTER TRIALS

Please refer to the reporting requirements in Policy 801: Reporting Research Events and Policy 802: Non-compliance in Human Subjects Research, found here. The participating site PI must immediately report to the IRB any deaths possibly related to the research within 24 hours of PI awareness of the event via the multi-site enhancement module. The participating site PI must also report any other events required by Policy 801 to the NIH IRB PI within 7 days of PI awareness.

Please also notify the coordinating center PI and study coordinator of your submission at the time you make it.

For IND studies, the site PI will also directly submit reports to the CCR as IND sponsor per section **8.3**.

7.4 NIH REQUIRED DATA AND SAFETY MONITORING PLAN

7.4.1 Principal Investigator/Research Team

The clinical research team will meet on a regular basis (weekly) when participants are being actively treated on the trial to discuss each participant.

All data will be collected in a timely manner and reviewed by the principal investigator. Events meeting requirements for expedited reporting as described in section **7.2.1** will be submitted within the appropriate timelines.

The principal investigator will review adverse event on each participant to ensure safety and data accuracy. The principal investigator will personally conduct or supervise the investigation and provide appropriate delegation of responsibilities to other members of the research staff.

8 SPONSOR PROTOCOL/SAFETY REPORTING

8.1 DEFINITIONS

8.1.1 Adverse Event

Any untoward medical occurrence in a participant or clinical investigation subject administered a pharmaceutical product and which does not necessarily have a causal relationship with this treatment. An adverse event (AE) can therefore be any unfavorable and unintended sign (including an abnormal laboratory finding), symptom, or disease temporally associated with the use of a medicinal (investigational) product, whether or not related to the medicinal (investigational) product (ICH E6 (R2)).

8.1.2 Serious Adverse Event (SAE)

An adverse event or suspected adverse reaction is considered serious if in the view of the investigator or the sponsor, it results in any of the following:

- Death,
- A life-threatening adverse event (see section **8.1.3**)
- Inpatient hospitalization or prolongation of existing hospitalization
    - A hospitalization/admission that is pre-planned (i.e., elective or scheduled surgery arranged prior to the start of the study), a planned hospitalization for pre-existing condition, or a procedure required by the protocol, without a serious deterioration in health, is not considered a serious adverse event.
    - A hospitalization/admission that is solely driven by non-medical reasons (e.g., hospitalization for participant convenience) is not considered a serious adverse event.
    - Emergency room visits or stays in observation units that do not result in admission to the hospital would not be considered a serious adverse event. The reason for seeking medical care should be evaluated for meeting one of the other serious criteria.
- Persistent or significant incapacity or substantial disruption of the ability to conduct normal life functions
- A congenital anomaly/birth defect
- Important medical events that may not result in death, be life-threatening, or require hospitalization may be considered a serious adverse drug experience when, based upon appropriate medical judgment, they may jeopardize the participant or subject and may require medical or surgical intervention to prevent one of the outcomes listed in this definition.

8.1.3 Life-threatening

An adverse event or suspected adverse reaction is considered "life-threatening" if, in the view of either the investigator or sponsor, its occurrence places the participant or subject at immediate risk of death. It does not include an adverse event or suspected adverse reaction that, had it occurred in a more severe form, might have caused death. (21CFR312.32).

8.1.4 Severity

The severity of each Adverse Event will be assessed utilizing the CTCAE version 5.

8.1.5 Relationship to Study Product

All AEs will have their relationship to study product assessed using the terms: related or not related.

- Related – There is a reasonable possibility that the study product caused the adverse event. Reasonable possibility means that there is evidence to suggest a causal relationship between the study product and the adverse event.
- Not Related – There is not a reasonable possibility that the administration of the study product caused the event.

8.2 ASSESSMENT OF SAFETY EVENTS

AE information collected will include event description, date of onset, assessment of severity and relationship to study product and alternate etiology (if not related to study product), date of resolution of the event, seriousness and outcome. The assessment of severity and relationship to the study product will be done only by those with the training and authority to make a diagnosis and listed on the Form FDA 1572 as the site principal investigator or sub-investigator. AEs occurring during the collection and reporting period will be documented appropriately regardless of relationship. AEs will be followed through resolution.

SAEs will be:

- Assessed for severity and relationship to study product and alternate etiology (if not related to study product) by a licensed study physician listed on the Form FDA 1572 as the site principal investigator or sub-investigator.
- Recorded on the appropriate SAE report form, the medical record and captured in the clinical database.
- Followed through resolution by a licensed study physician listed on the Form FDA 1572 as the site principal investigator or sub-investigator.

For timeframe of recording adverse events, please refer to section **6.1**. All serious adverse events recorded from the time of first investigational product administration must be reported to the sponsor with the exception of any listed in section **8.4**.

8.3 REPORTING OF SERIOUS ADVERSE EVENTS

Any AE that meets protocol-defined serious criteria that require expedited reporting must be submitted immediately (within 24 hours of awareness) to OSRO Safety using the CCR SAE report form.

All SAE reporting must include the elements described in section **8.2**.

SAE reports will be submitted to the Center for Cancer Research (CCR) at: OSROSafety@mail.nih.gov and to the CCR PI and study coordinator. CCR SAE report form and instructions can be found at: https://ccrod.cancer.gov/confluence/display/CCRCRO/Forms+and+Instructions

Following the assessment of the SAE by OSRO, other supporting documentation of the event may be requested by the OSRO Safety and should be provided as soon as possible.

8.4 WAIVER OF EXPEDITED REPORTING TO CCR

Death or hospitalization that is deemed to be due to disease progression, and not attributable to the intervention will not be reported as an SAE. The event, and the assessment that it was caused by disease progression will be documented in the medical records. The causality assessment of Hospitalization will be re-evaluated any time when new information is received. If the causality assessment changes from disease progression to related to the study intervention, SAE report will be sent to the Sponsor immediately in an expedited manner according to section **8.3**. If there is any uncertainty whether the intervention is a contributing factor to the event, the event should be reported as AE or SAE as appropriate.

8.5 REPORTING PREGNANCY

All required pregnancy reports/follow-up to OSRO will be submitted to: OSROSafety@mail.nih.gov and to the CCR PI and study coordinator.

Forms and instructions can be found here: https://ccrod.cancer.gov/confluence/display/CCRCRO/Forms+and+Instructions

8.5.1 Maternal Exposure

If a participant becomes pregnant while actively on study intervention, the study intervention should be discontinued immediately, and the pregnancy reported to the Sponsor no later than 24 hours of when the Investigator becomes aware of it. The Investigator should notify the Sponsor no later than 24 hours of when the outcome of the Pregnancy becomes known.

Pregnancy itself is not regarded as an SAE. However, congenital abnormalities or birth defects and spontaneous miscarriages that meet serious criteria (section **8.1.2**) should be reported as SAEs.

The outcome of all pregnancies (spontaneous miscarriage, elective termination, ectopic pregnancy, normal birth, or congenital abnormality) occurring within 2 months of last ^{18}F-DCFPyL imaging should be followed up and documented.

8.5.2 Paternal Exposure

Male participants should refrain from fathering a child or donating sperm during the study and for 2 months after the last dose of ^{18}F-DCFPyL.

Pregnancy of the participant's partner is not considered to be an AE. However, the outcome of all pregnancies (spontaneous miscarriage, elective termination, ectopic pregnancy, normal birth, or congenital abnormality) occurring from the date of the first dose until 2 months after the last imaging should, if possible, be followed up and documented.

8.6 REGULATORY REPORTING FOR STUDIES CONDUCTED UNDER CCR SPONSORED IND

Following notification from the investigator, CCR, the IND sponsor, will report any suspected adverse reaction that is both serious and unexpected. CCR will report an AE as a suspected adverse reaction only if there is evidence to suggest a causal relationship between the study product and the adverse event. CCR will notify FDA and all participating investigators (i.e., all investigators to whom the sponsor is providing drug under its INDs or under any investigator's

IND) in an IND safety report of potential serious risks from clinical trials or any other source, as soon as possible, in accordance to 21 CFR Part 312.32.

All serious events will be reported to the FDA at least annually in a summary format.

8.7 Sponsor Protocol Deviation Reporting

A Protocol Deviation is defined as any non-compliance with the clinical trial Protocol, Manual of Operational Procedures (MOP) and other Sponsor approved study related documents, GCP, or protocol-specific procedural requirements on the part of the participant, the Investigator, or the study site staff inclusive of site personnel performing procedures or providing services in support of the clinical trial.

It is the responsibility of the study Staff to document any protocol deviation identified by the Staff or the site Monitor in the CCR Protocol Deviation Tracking System (PDTS) online application. The entries into the PDTS online application should be timely, complete, and maintained per CCR PDTS user requirements.

In addition, any deviation to the protocol should be documented in the participant's source records and reported to the reviewing IRB per their guidelines. OSRO required protocol deviation reporting is consistent with E6(R2) GCP: Integrated Addendum to ICH E6(R1): 4.5 Compliance with Protocol; 5.18.3 (a), and 5.20 Noncompliance; and ICH E3 16.2.2 Protocol deviations.

9 CLINICAL MONITORING PLAN

Clinical site monitoring is conducted to ensure:

- that the rights of the participants are protected;
- that the study is implemented per the approved protocol, Good Clinical Practice and standard operating procedures; and,
- the quality and integrity of study data and data collection methods are maintained.

Monitoring for this study will be performed by NCI CCR Office of Sponsor and Regulatory Oversight (OSRO) and Regulatory Oversight Support (SROS) Services contractor. Clinical site monitoring activities will be based on OSRO standards, FDA Guidance E6(R2) Good Clinical Practice: Integrated Addendum to ICH E6(R1) March 2018, and applicable regulatory requirements.

Details of clinical site monitoring will be documented in a Clinical Monitoring Plan (CMP) developed by OSRO. CMPs will be protocol-specific, risk-based and tailored to address human subject protections and integrity of the study data. OSRO will determine the intensity and frequency of monitoring based on several factors, including study type, phase, risk, complexity, expected enrollment rate, and any unique attributes of the study and the site. The Sponsor will conduct a periodic review of the CMP to confirm the plan's continued appropriateness. A change to the protocol, significant or pervasive non-compliance with GCP, or the protocol may trigger CMP updates.

OSRO SROS Monitoring visits and related activities will be conducted throughout the life cycle of each protocol. The first activity is before the study starts to conduct a Site Assessment Visit

(SAV) (as warranted), followed by a Site Initiation Visit (SIV), Interim Monitoring Visit(s) (IMVs), and a study Close-Out Visit (COV).

Some monitoring activities may be performed remotely, while others will occur at the study site(s). Monitoring visit reports will describe visit activities, observations, and associated action items or follow-up required for resolution of any issues, discrepancies, or deviations. Monitoring reports will be distributed to the study PI, NCI CCR QA, CCR Protocol Support Office, coordinating center (if applicable), and the Sponsor regulatory file.

The site Monitor will inform the study team of any deviations observed during monitoring visits. If unresolved, the Monitor will request that the site Staff enter the deviations in the CCR Protocol Deviation Tracking System (PDTS) for deviation reporting to the Sponsor and as applicable per institutional and IRB guidance.

10 STATISTICAL CONSIDERATIONS

10.1 PRIMARY ENDPOINT

^{18}F-DCFPyL PET/CT will be used to identify positive suspected sites, which will be correlated with histopathology results. For lesions within the liver, a focal abnormal area of increased ^{18}F-DCFPyL activity higher than the surrounding liver uptake (SUVmax more than x 1.2 times than the normal liver-SUV mean) will be considered positive. For lesions outside the liver, a positive lesion is defined as focal abnormal uptake higher than the blood pool, or the surrounding normal organ or soft tissue background. Pathological anatomic imaging but lacking DCFPyL uptake would indicate a type of tumor that fails to express PSMA. The performance of ^{18}F-DCFPyL PET/CT is assessed by the positive predictive value which is defined as the proportion of histopathology positive lesions.

10.2 SECONDARY ENDPOINTS

- Compare the lesion level sensitivity, specificity and positive predictive value of ^{18}F-DCFPyL PET/CT with standard CT and/or MRI imaging, and ^{18}F-FDG PET/CT.
- Compare ^{18}F-DCFPyL PET/CT uptake between pre- and post-treatment in ^{18}F-DCFPyL PET/CT positive participants (as measured by change in pre-treatment and post-treatment SUV) to detect treatment response following local treatment.

10.3 EXPLORATORY ENDPOINTS

- Correlate the intensity of uptake of ^{18}F-DCFPyL PET/CT with respect to the CT and/or MRI tumor grade.
- Correlate tumor tissue histological PSMA expression with the intensity of uptake of ^{18}F-DCFPyL PET/CT.
- Compare tumor viability and metabolism determined by ^{18}F-FDG PET imaging with ^{18}F-DCFPyL uptake.
- ^{18}F-DCFPyL intensity of uptake (expressed as the maximum standard uptake value – SUVmax) and tumor volume will be correlated with 5 year overall survival, and 5 year progression free survival.

- Evaluate tumor biomarkers in blood, urine and tissue samples.

10.4 Sample Size Determination

Sample size was calculated such that the limits of the 95% expected confidence interval to the true lesion level positive predicted value is less than 15%. The lesion level positive predictive value is the proportion of biopsy positive lesions. Assume 80% of the participants enrolled to the study each have 1-2 lesions identified by ^{18}F-DCFPyL PET/CT with 0.2 modest inter-lesion correlation, and the positive predictive value is 70%. A sample size of 40 evaluable participants accrued to the study produces a two-sided 95% confidence interval with the distance from the positive predictive value to the limits equal to 0.148. An evaluable participant is defined as a participant who completes all required study procedures. To account for inevaluable participants, the accrual ceiling will be set to 50.

10.5 Analysis of the Primary Endpoint

The point estimates and 95% confidence intervals of the positive predictive value of ^{18}F-DCFPyL PET/CT will be reported in which the confidence limits are the 2.5th and 97.5th percentile of the 2000 bootstrap samples obtained by random sample without replacement at the participant level to account for inter-lesion correlation.

10.6 Analysis of the Secondary Endpoints

- The lesion level sensitivity, specificity and positive predictive value of ^{18}F-DCFPyL PET/CT and CT/MRI will be calculated and compared. The confidence interval for each estimate will be obtained from the bootstrap samples as described in **10.5** and the difference in the estimates between the imaging modalities will be compared by the Wald test with the standard error calculated from the bootstrap samples.
- For ^{18}F-DCFPyL PET/CT positive participants who undergo local treatment for HCC, change in ^{18}F-DCFPyL PET/CT uptake between pre- and post-treatment of tumor or tumor bed will be compared by paired Wilcoxon test.

10.7 Analysis of Exploratory endpoints

- The uptake of ^{18}F-DCFPyL PET/CT will be correlated with CT/MRI grade by Kendall's tau-b correlation.
- The uptake of ^{18}F-DCFPyL PET/CT will be correlated with tumor tissue histological PSMA expression by Kendall's tau-b correlation.
- The uptake of ^{18}F-DCFPyL PET/CT measured by maximum Standard Uptake Value (SUV) will be correlated with ^{18}F-FDG uptake by Spearman rank correlation. These SUV uptake measurements will also be correlated with PSMA expression as determined by standard histological assessment of tumor tissue.
- Uptake of ^{18}F-DCFPyL PET/CT with be correlated with 5 year progression free survival (via scans collected from local providers during routine follow up) and 5 year overall survival by Cox regression analysis.
- Descriptive statistics will be used to summarize tumor biomarkers in blood and tissue samples.

10.8 Interim Futility Analysis and Early Stopping Rule

To avoid excessive expense and radiation exposure, if ^{18}F-DCFPyL PET/CT is not successful in identifying lesions, we will implement the following interim futility analysis and early stopping rule.

We plan to stop the protocol if the first 5 participants with lesions greater than 1 cm have negative DCFPyL uptake (defined as tumor uptake less than adjacent background soft tissue, or less than blood pool for lymph nodes).

11 COLLABORATIVE AGREEMENTS

11.1 Transfers associated with correlative studies conducted under an approved protocol

Investigators in the NIH intramural program may participate in multi-site clinical trials (either as a site or as the coordinating center) under which human materials are transferred from the intramural program to another site for correlative studies that are part of the approved protocol. In such a situation, the protocol clearly documents the tests conducted under the correlative studies, and each institution participating in the clinical study is bound by the terms of their Protocol and their obligations under the statutes and regulations. In addition, intramural protocols are cleared by the IC Clinical Director. In such situations, use of an HM-MTA is not necessary for these transfers.

11.2 Multi-Institutional Guidelines

11.2.1 Sites Reviewed by NIH Intramural IRB

After initial approval, participating sites can submit the following: site-specific amendments and reportable events to the NIH IRB via the multi-site enhancement module. Before reaching the NIH IRB, each participating site submission gets routed to the NIH coordinating center for sign-off. If accepted, the action is submitted to the NIH IRB.

12 HUMAN SUBJECTS PROTECTIONS

12.1 Rationale For Subject Selection

All participants with HCC meeting eligibility criteria may participate in this study. Individuals of any race, ethnic group, or gender will be eligible for this study. Physically impaired persons who otherwise satisfy eligibility criteria will be included in this study.

12.2 Participation of Children

Children will not be considered as research subjects for this study as ^{18}F-DCFPyL dosing and toxicity has not been established in this population.

12.3 EVALUATION OF BENEFITS AND RISKS/DISCOMFORTS

12.3.1 Risk/Benefit Assessment

12.3.1.1 Known Potential Risks

12.3.1.2 Risks related to ^{18}F-DCFPyL

The risks related to ^{18}F-DCFPyL include radiation exposure allergic reaction, fatigue, dysgeusia, and headache.

12.3.1.3 Risks related to ^{18}F-FDG

Risks associated with ^{18}F-FDG include radiation and hypersensitivity reaction.

12.3.1.4 Risks related to IV

The risks associated with IV include temporary pain, bleeding and bruising at the infusion site, leakage of ^{18}F-DCFPyL and ^{18}F-FDG into the skin and tissue around the IV and infection.

12.3.1.5 Risks related to PET/CT scan

The risks associated PET/CTs include IV insertion site pain, bruising, dizziness, hypotension, inflammation of the vein or infection at the needle site. Other side effects could include discomfort from lying on a hard surface for an extended period and infection at the IV site or blood.

12.3.1.5.1 Risks of exposure to Ionizing Radiation

This research study involves exposure to radiation from up to 3 ^{18}F-DCFPyL PET/CTs, 3 regional CTs of a single field-of-view including the liver lesion (for the dynamic portion of the ^{18}F-DCFPyL PET/CT), up to 3 ^{18}F-FDG PET/CTs, up to 3 chest/abdomen/pelvis CTs, and 2 CT-guided biopsies. The amount of radiation exposure from these procedures is equal to approximately 12.6 rem. This amount is more than would be expected from everyday background radiation. Being exposed to excess radiation can increase the risk of cancer. The risk of getting cancer from the radiation exposure in this study is 1.3 out of 100 (1.3%) and of getting a fatal cancer is 0.6 out of 100 (0.6%).

Note: dosimetry estimates are based on NIH scanners. Estimates at participating sites may vary.

12.3.1.6 CT contrast risk

Itching, hives, shortness of breath, shock and rarely death are possible risks associated with contrast agents that may be used during CT imaging. Very rarely, the contrast agents used in CT can cause kidney problems for certain participants, such as those with impaired kidney function.

12.3.1.7 MRI and MRI Contrast Risks

Participants are at risk for injury from the MRI magnet if they have metal in their body. There is a possibility that participants may experience claustrophobia. There are risks of back discomfort related to lying in the scanner.

The most common side effects from MRI contrast (gadolinium) include injection site pain, metallic taste, nausea, hives, headaches, difficulty breathing, hypotension and an allergic reaction. Serious but rare side effects such as gadolinium toxicity and nephrogenic systemic fibrosis, or NSF, are most often seen in patients with severe kidney problems.

The risks of an IV catheter include bleeding, infection, or inflammation, pain and swelling of the skin and vein.

12.3.1.8 Risks of Blood Draw

Side effects of blood draws include pain and bruising in the area where the needle was placed, lightheadedness, and rarely, fainting. Up to 17 mL of blood maybe collected at each study visit when a ^{18}F-DCFPyL PET/CT occurs. Up to 7 mL of blood may be collected at each follow-up visit.

12.3.1.9 Risk of Biopsy

All care will be taken to minimize risks that may be incurred by tumor sampling. Procedure-related risks include pain, bleeding, tenderness, scarring, and infection. Complications from biopsies may be life threatening and may include organ damage.

There is a possibility that conscious sedation may be used for the procedure. The common side effects of conscious sedation include drowsiness, delayed reflexes, hypotension, headache, and nausea. These are generally mild and last no more than a few hours.

12.3.1.10 Risk from Urine Collection

There are no known risks associated with urine collection.

12.3.1.11 Known Potential Benefits

There are no direct benefits to the study participant; however, the study results may help the investigators learn more about the effectiveness of ^{18}F-DCFPyL imaging in identifying sites of HCC and could help confirm treatment success.

12.4 Consent Process and Documentation

The informed consent document will be provided as a physical or electronic document to the participant as applicable for review prior to consenting. A designated study investigator will carefully explain the procedures and tests involved in this study, and the associated risks, discomforts and benefits. In order to minimize potential coercion, as much time as is needed to review the document will be given, including an opportunity to discuss it with friends, family members and/or other advisors, and to ask questions of any designated study investigator. A signed informed consent document will be obtained prior to entry onto the study.

The initial consent process as well as re-consent, when required, may take place in person or remotely (e.g., via telephone or other NIH approved remote platforms used in compliance with local policy, and/including HRPP Policy 303) per discretion of the designated study investigator and with the agreement of the participant. Whether in person or remote, the privacy of the subject will be maintained. Consenting investigators (and participant, when in person) will be located in a private area (e.g., clinic consult room). When consent is conducted remotely, the participant will be informed of the private nature of the discussion and will be encouraged to relocate to a more private setting if needed.

Consent will be documented with required signatures on the physical document (which includes the printout of an electronic document sent to participant) or on the electronic document, with a manual (non-electronic) signature on the electronic document. When required, witness signature will be obtained similarly as described for the investigator and participant.

Please see site specific supplement for electronic signature requirements for each site.

13 REGULATORY AND OPERATIONAL CONSIDERATIONS

13.1 Study Discontinuation and Closure

This study may be temporarily suspended or prematurely terminated if there is sufficient reasonable cause. Written notification, documenting the reason for study suspension or termination, will be provided by the suspending or terminating party to study participants, investigator, funding agency, the Investigational New Drug (IND) sponsor and regulatory authorities. If the study is prematurely terminated or suspended, the Principal Investigator (PI) will promptly inform study participants, the Institutional Review Board (IRB), and sponsor and will provide the reason(s) for the termination or suspension. Study participants will be contacted, as applicable, and be informed of changes to study visit schedule.

Circumstances that may warrant termination or suspension include, but are not limited to:

- Determination of unexpected, significant, or unacceptable risk to participants
- Demonstration of efficacy that would warrant stopping
- Insufficient compliance to protocol requirements
- Data that are not sufficiently complete and/or evaluable
- Determination that the primary endpoint has been met
- Determination of futility

Study may resume once concerns about safety, protocol compliance, and data quality are addressed, and satisfy the sponsor, IRB and as applicable, Food and Drug Administration (FDA).

13.2 Quality Assurance and Quality Control

Each clinical site will perform internal quality management of study conduct, data and biological specimen collection, documentation and completion. An individualized quality management plan will be developed to describe a site's quality management.

Quality control (QC) procedures will be implemented beginning with the data entry system and data QC checks that will be run on the database will be generated. Any missing data or data anomalies will be communicated to the site(s) for clarification/resolution.

Following written Standard Operating Procedures (SOPs), the monitors will verify that the clinical trial is conducted and data are generated and biological specimens are collected, documented (recorded), and reported in compliance with the protocol, International Conference on Harmonisation Good Clinical Practice (ICH GCP), and applicable regulatory requirements (e.g., Good Laboratory Practices (GLP), Good Manufacturing Practices (GMP)).

The investigational site will provide direct access to all trial related sites, source data/documents, and reports for the purpose of monitoring and auditing by the sponsor, and inspection by local and regulatory authorities.

13.3 Conflict of Interest Policy

The independence of this study from any actual or perceived influence, such as by the pharmaceutical industry, is critical. Therefore, any actual conflict of interest of persons who have a role in the design, conduct, analysis, publication, or any aspect of this trial will be

disclosed and managed. Furthermore, persons who have a perceived conflict of interest will be required to have such conflicts managed in a way that is appropriate to their participation in the design and conduct of this trial. The study leadership in conjunction with the NCI has established policies and procedures for all study group members to disclose all conflicts of interest and will establish a mechanism for the management of all reported dualities of interest.

13.4 CONFIDENTIALITY AND PRIVACY

Participant confidentiality and privacy is strictly held in trust by the participating investigators, their staff, and the sponsor(s). This confidentiality is extended to cover testing of biological samples and genetic tests in addition to the clinical information relating to participants. Therefore, the study protocol, documentation, data, and all other information generated will be held in strict confidence. No information concerning the study or the data will be released to any unauthorized third party without prior written approval of the sponsor.

All research activities will be conducted in as private a setting as possible.

The study monitor, other authorized representatives of the sponsor, representatives of the Institutional Review Board (IRB), and/or regulatory agencies may inspect all documents and records required to be maintained by the investigator, including but not limited to, medical records (office, clinic, or hospital) and pharmacy records for the participants in this study. The clinical study site will permit access to such records.

The study participant's contact information will be securely stored at the/each clinical site for internal use during the study. At the end of the study, all records will continue to be kept in a secure location for as long a period as dictated by the reviewing IRB, Institutional policies, or sponsor requirements.

Study participant research data, which is for purposes of statistical analysis and scientific reporting, will be transmitted to and stored at the NCI CCR. This will not include the participant's contact or identifying information. Rather, individual participants and their research data will be identified by a unique study identification number. The study data entry and study management systems used by the clinical site(s) and by NCI CCR research staff will be secured and password protected. At the end of the study, all study databases will be archived at the NIH.

To further protect the privacy of study participants, a Certificate of Confidentiality has been issued by the National Institutes of Health (NIH). This certificate protects identifiable research information from forced disclosure. It allows the investigator and others who have access to research records to refuse to disclose identifying information on research participation in any civil, criminal, administrative, legislative, or other proceeding, whether at the federal, state, or local level. By protecting researchers and institutions from being compelled to disclose information that would identify research participants, Certificates of Confidentiality help achieve the research objectives and promote participation in studies by helping assure confidentiality and privacy to participants.

14 PHARMACEUTICAL INFORMATION/COMMERCIAL IMAGING

14.1 ^{18}F-DCFPyL (IND: 133631)

14.1.1 Source/Acquisition and Accountability

14.1.1.1 Agent Ordering

^{18}F-DCFPyL will be ordered from NIH PET department. MIB staff will contact the radiopharmacy, a date and time will be assigned, and an order placed with the radiopharmacy using the NIH Supply ordering system for Radiopharmaceuticals. http://supply.cc.nih.gov/.

14.1.1.2 Agent returns

The investigator or appropriate investigator-designee will order subject doses of the IND agent for this specific trial. The investigational radiopharmaceutical will be shipped to the site on the day the participant is to be injected, taking into account varying radioactive half-lives for different radioactive imaging agents.

14.1.1.3 Agent Inventory Records

If for any reason the study imaging is unable to be completed, sites will allow the radioactivity of the ^{18}F-DCFPyL solution to decay and then discard it appropriately per site's policies and procedures, making a record of the event as required. A copy of the policy should be available upon request.

14.1.2 Toxicity

Refer to section **1.2.2**.

14.1.3 Formulation and Preparation

No preparation required by study staff and/or study participants. The ^{18}F-DCFPyL used in this study is prepared in the PET Department CC/NIH. ^{18}F-DCFPyL for each study participant will be received in individual participant doses from the PET department. Containers that are radioactive or contain radioactive products will be disposed of per NIH Radiation Safety Guidelines.

Manufacture of ^{18}F-DCFPyL drug substance and formulation, sterilization and filling of ^{18}F-DCFPyL Injection drug product, is a continuous process whereby the drug substance is never isolated or held. Immediately upon completion of the radiosynthesis reaction that forms ^{18}F-DCFPyL drug substance, the reaction mixture is purified by high performance liquid chromatography and formulated in a solution of Sodium Chloride Injection (0.9%) containing ethanol. A sample is removed for analytical testing under aseptic conditions for chemical and radiochemical quality control analysis, and bacterial endotoxin and sterility testing. This completes the manufacture of ^{18}F-DCFPyL injection. The lowest limit for specific-activity (SA) that will be used at the time of injection is 1000 mCi/µmole, although the validation and phase I study radiosynthesis SA was significantly higher than 1000 mCi/µmole.

14.1.4 Stability and Storage

The in-use shelf-life of ^{18}F-DCFPyL will be specified on the label. Although from a chemical perspective, the product remains stable beyond 6 hours, due to the short 109.8-minute half-life of ^{18}F, the low level of activity present after 6 hours renders it unsuitable for positron imaging

tomography studies. ^{18}F-DCFPyL is stored in the original container at 4 °C under inert atmosphere.

14.1.5 Administration Procedures

Refer to section **3.2**.

14.2 ^{18}F-FDG (IND: 133631)

For complete information, refer to package insert.

14.2.1 Source/Acquisition and Accountability

Commercial supplies of ^{18}F-FDG will be purchased for use in this study.

14.2.2 Administration Procedures

Refer to section **3.2.3**.

14.3 COMMERCIAL IMAGING

The imaging devices used as comparators on this study (CT scanner and MRI) are exempt as they meet 21CFR 812.2 (c) criteria for exemption under category 2 in that they are being used in accordance with labeling.

15 REFERENCES

1. Chen Y, Pullambhatla M, Foss CA, Byun Y, Nimmagadda S, Senthamizhchelvan S, Sgouros G, Mease RC, Pomper MG: **2-(3-{1-Carboxy-5-[(6-[18F]fluoro-pyridine-3-carbonyl)-amino]-pentyl}-ureido)-pen tanedioic acid, [18F]DCFPyL, a PSMA-based PET imaging agent for prostate cancer**. *Clinical cancer research : an official journal of the American Association for Cancer Research* 2011, **17**(24):7645-7653.
2. Szabo Z, Mena E, Rowe SP, Plyku D, Nidal R, Eisenberger MA, Antonarakis ES, Fan H, Dannals RF, Chen Y *et al*: **Initial Evaluation of [(18)F]DCFPyL for Prostate-Specific Membrane Antigen (PSMA)-Targeted PET Imaging of Prostate Cancer**. *Molecular imaging and biology : MIB : the official publication of the Academy of Molecular Imaging* 2015, **17**(4):565-574.
3. Gorin MA, Rowe SP, Patel HD, Vidal I, Mana-Ay M, Javadi MS, Solnes LB, Ross AE, Schaeffer EM, Bivalacqua TJ *et al*: **Prostate Specific Membrane Antigen Targeted (18)F-DCFPyL Positron Emission Tomography/Computerized Tomography for the Preoperative Staging of High Risk Prostate Cancer: Results of a Prospective, Phase II, Single Center Study**. *J Urol* 2018, **199**(1):126-132.
4. Rowe SP, Campbell SP, Mana-Ay M, Szabo Z, Allaf ME, Pienta KJ, Pomper MG, Ross AE, Gorin MA: **Prospective Evaluation of PSMA-Targeted (18)F-DCFPyL PET/CT in Men with Biochemical Failure after Radical Prostatectomy for Prostate Cancer**. *J Nucl Med* 2019.
5. Rowe SP, Macura KJ, Mena E, Blackford AL, Nadal R, Antonarakis ES, Eisenberger M, Carducci M, Fan H, Dannals RF *et al*: **PSMA-Based [(18)F]DCFPyL PET/CT Is Superior to Conventional Imaging for Lesion Detection in Patients with Metastatic Prostate Cancer**. *Molecular imaging and biology : MIB : the official publication of the Academy of Molecular Imaging* 2016, **18**(3):411-419.
6. Rowe SP, Li X, Trock BJ, Werner RA, Frey SM, DiGianvittorio M, Bleiler K, Reyes DK, Abdallah R, Pienta KJ *et al*: **Prospective Comparison of PET Imaging with PSMA-targeted (18)F-DCFPyL versus Na(18)F for Bone Lesion Detection in Patients with Metastatic Prostate Cancer**. *J Nucl Med* 2019.
7. Szabo Z, Mena E, Rowe SP, Plyku D, Nidal R, Eisenberger MA, Antonarakis ES, Fan H, Dannals RF, Chen Y *et al*: **Initial Evaluation of [F]DCFPyL for Prostate-Specific Membrane Antigen (PSMA)-Targeted PET Imaging of Prostate Cancer**. *Molecular imaging and biology : MIB : the official publication of the Academy of Molecular Imaging* 2015.
8. Mena E, Lindenberg ML, Turkbey IB, Shih JH, Harmon SA, Lim I, Lin F, Adler S, Eclarinal P, McKinney YL *et al*: **(18)F-DCFPyL PET/CT Imaging in Patients with Biochemically Recurrent Prostate Cancer After Primary Local Therapy**. *J Nucl Med* 2020, **61**(6):881-889.
9. Gaur S, Mena E, Harmon SA, Lindenberg ML, Adler S, Ton AT, Shih JH, Mehralivand S, Merino MJ, Wood BJ *et al*: **Prospective Evaluation of (18)F-DCFPyL PET/CT in Detection of High-Risk Localized Prostate Cancer: Comparison With mpMRI**. *AJR Am J Roentgenol* 2020:1-8.

10. Rousseau E, Wilson D, Lacroix-Poisson F, Krauze A, Chi K, Gleave M, McKenzie M, Tyldesley S, Goldenberg SL, Benard F: **A Prospective Study on (18)F-DCFPyL PSMA PET/CT Imaging in Biochemical Recurrence of Prostate Cancer**. *J Nucl Med* 2019, **60**(11):1587-1593.

11. Ghosh A, Heston WD: **Tumor target prostate specific membrane antigen (PSMA) and its regulation in prostate cancer**. *J Cell Biochem* 2004, **91**(3):528-539.

12. Mannweiler S, Amersdorfer P, Trajanoski S, Terrett JA, King D, Mehes G: **Heterogeneity of prostate-specific membrane antigen (PSMA) expression in prostate carcinoma with distant metastasis**. *Pathol Oncol Res* 2009, **15**(2):167-172.

13. Ross JS, Sheehan CE, Fisher HA, Kaufman RP, Jr., Kaur P, Gray K, Webb I, Gray GS, Mosher R, Kallakury BV: **Correlation of primary tumor prostate-specific membrane antigen expression with disease recurrence in prostate cancer**. *Clinical cancer research : an official journal of the American Association for Cancer Research* 2003, **9**(17):6357-6362.

14. Backhaus P, Noto B, Avramovic N, Grubert LS, Huss S, Bogemann M, Stegger L, Weckesser M, Schafers M, Rahbar K: **Targeting PSMA by radioligands in non-prostate disease-current status and future perspectives**. *Eur J Nucl Med Mol Imaging* 2018, **45**(5):860-877.

15. Nimmagadda S, Pullambhatla M, Chen Y, Parsana P, Lisok A, Chatterjee S, Mease R, Rowe SP, Lupold S, Pienta KJ *et al*: **Low-Level Endogenous PSMA Expression in Nonprostatic Tumor Xenografts Is Sufficient for In Vivo Tumor Targeting and Imaging**. *J Nucl Med* 2018, **59**(3):486-493.

16. Bauman G, Martin P, Thiessen JD, Taylor R, Moussa M, Gaed M, Rachinsky I, Kassam Z, Chin J, Pautler S *et al*: **[(18)F]-DCFPyL Positron Emission Tomography/Magnetic Resonance Imaging for Localization of Dominant Intraprostatic Foci: First Experience**. *Eur Urol Focus* 2018, **4**(5):702-706.

17. Mittal S, El-Serag HB: **Epidemiology of hepatocellular carcinoma: consider the population**. *J Clin Gastroenterol* 2013, **47 Suppl**:S2-6.

18. European Association for the Study of the Liver. Electronic address eee, European Association for the Study of the L: **EASL Clinical Practice Guidelines: Management of hepatocellular carcinoma**. *J Hepatol* 2018, **69**(1):182-236.

19. Agnello F, Salvaggio G, Cabibbo G, Maida M, Lagalla R, Midiri M, Brancatelli G: **Imaging appearance of treated hepatocellular carcinoma**. *World J Hepatol* 2013, **5**(8):417-424.

20. Li YC, Yang CS, Zhou WL, Li HS, Han YJ, Wang QS, Wu HB: **Low glucose metabolism in hepatocellular carcinoma with GPC3 expression**. *World J Gastroenterol* 2018, **24**(4):494-503.

21. Asman Y, Evenson AR, Even-Sapir E, Shibolet O: **[18F]fludeoxyglucose positron emission tomography and computed tomography as a prognostic tool before liver transplantation, resection, and loco-ablative therapies for hepatocellular carcinoma**. *Liver Transpl* 2015, **21**(5):572-580.

22. Zhu H, Shao Q, Sun X, Deng Z, Yuan X, Yu D, Zhou X, Ding Y: **The mobilization, recruitment and contribution of bone marrow-derived endothelial progenitor cells to the tumor neovascularization occur at an early stage and throughout the entire process of hepatocellular carcinoma growth**. *Oncol Rep* 2012, **28**(4):1217-1224.

23. Jiao D, Li Y, Yang F, Han D, Wu J, Shi S, Tian F, Guo Z, Xi W, Li G *et al*: **Expression of Prostate-Specific Membrane Antigen in Tumor-Associated Vasculature Predicts Poor Prognosis in Hepatocellular Carcinoma**. *Clin Transl Gastroenterol* 2019.

24. Tolkach Y, Goltz D, Kremer A, Ahmadzadehfar H, Bergheim D, Essler M, Lam M, de Keizer B, Fischer HP, Kristiansen G: **Prostate-specific membrane antigen expression in hepatocellular carcinoma: potential use for prognosis and diagnostic imaging**. *Oncotarget* 2019, **10**(41):4149-4160.

25. Patel D, Loh H, Le K, Stevanovic A, Mansberg R: **Incidental Detection of Hepatocellular Carcinoma on 68Ga-Labeled Prostate-Specific Membrane Antigen PET/CT**. *Clin Nucl Med* 2017, **42**(11):881-884.

26. Kesler M, Levine C, Hershkovitz D, Mishani E, Menachem Y, Lerman H, Zohar Y, Shibolet O, Even-Sapir E: **(68)Ga-PSMA is a novel PET-CT tracer for imaging of hepatocellular carcinoma: A prospective pilot study**. *J Nucl Med* 2018.

27. Kuyumcu S, Has-Simsek D, Iliaz R, Sanli Y, Buyukkaya F, Akyuz F, Turkmen C: **Evidence of Prostate-Specific Membrane Antigen Expression in Hepatocellular Carcinoma Using 68Ga-PSMA PET/CT**. *Clin Nucl Med* 2019, **44**(9):702-706.

28. Dietlein M, Kobe C, Kuhnert G, Stockter S, Fischer T, Schomacker K, Schmidt M, Dietlein F, Zlatopolskiy BD, Krapf P *et al*: **Comparison of [(18)F]DCFPyL and [(68)Ga]Ga-PSMA-HBED-CC for PSMA-PET Imaging in Patients with Relapsed Prostate Cancer**. *Molecular imaging and biology : MIB : the official publication of the Academy of Molecular Imaging* 2015, **17**(4):575-584.

16 APPENDICES

16.1 APPENDIX A - PERFORMANCE STATUS CRITERIA

ECOG Performance Status Scale	
Grade	Descriptions
0	Normal activity. Fully active, able to carry on all pre-disease performance without restriction.
1	Symptoms, but ambulatory. Restricted in physically strenuous activity, but ambulatory and able to carry out work of a light or sedentary nature (e.g., light housework, office work).
2	In bed <50% of the time. Ambulatory and capable of all self-care, but unable to carry out any work activities. Up and about more than 50% of waking hours.
3	In bed >50% of the time. Capable of only limited self-care, confined to bed or chair more than 50% of waking hours.
4	100% bedridden. Completely disabled. Cannot carry on any self-care. Totally confined to bed or chair.
5	Dead.
