# Supplementary material for: Functional Imaging of Liver Cancer (FLIC): Study protocol of a phase 2 trial of 18F-DCFPyL PET/CT imaging for patients with hepatocellular carcinoma
Source: PLoS One. 2022 Nov 11;17(11):e0277407. doi: 10.1371/journal.pone.0277407 (PMC9651549; doi:10.1371/journal.pone.0277407)
Supplement: S1 File — (PDF) [file pone.0277407.s002.pdf]

NIH Intramural Research Program
Our Research Changes Lives

Memo Date: 02/23/2022

DOCUMENTATION OF COMPLETION OF NIH CONFLICT OF INTEREST REQUIREMENTS

It is the Federal Government's policy to eliminate or minimize actual or perceived conflict of interest in the conduct of non-exempt human subjects research. Consistent with 45 C.F.R. Part 46, NIH implements a number of laws, regulations, and policies regarding conflicts in non-exempt human subjects research. NIH employees engaged in agency work are subject to the comprehensive body of law governing the conduct of Federal employees. The applicable statutes and regulations include 18 U.S.C. §§ 201-216, the government-wide Standards of Ethical Conduct for Employees of the Executive Branch, 5 C.F.R. Parts 2634, 2635, and 2640, and agency-specific regulations (the Supplemental Standards of Ethical Conduct for Employees of the Department of Health and Human Services, 5 C.F.R. Part 5501). At all times, NIH employees are responsible for complying with all applicable ethical conduct rules, including those related to criminal conflict of interest (18 U.S.C. § 208), impartiality in the performance of official duties (5 C.F.R. § 2635.502), and financial disclosure (5 C.F.R. Part 2634), the agency has issued policies and guidelines to avoid conflicts of interest in clinical research.

Protocol Details

iRIS Reference Number: 569951

IRB Number: 000080

Protocol Title: 18F-DCFPyL PET/CT in Hepatocellular Carcinoma

NIH Principal/Lead Site Investigator(s): Freddy Escorcia

Protocol Action: Amendment

Determination

In connection with the protocol listed above, applicable NIH policies and guidelines to avoid conflicts of interest in non-exempt human subjects research have and will be followed for the duration of the protocol.

This protocol is subject to NIH conflicts of interest review and all related requirements have been met pursuant to Policy 102.

NIH Institute/Center (IC) Deputy Ethics Counselor (DEC) - NCI Committee.

NIH IC DEC Review (Approved by and Date):

Signature applied by Katherine Foss on 02/23/2022 04:14:36 PM EST
