# Supplementary material for: Functional Imaging of Liver Cancer (FLIC): Study protocol of a phase 2 trial of 18F-DCFPyL PET/CT imaging for patients with hepatocellular carcinoma
Source: PLoS One. 2022 Nov 11;17(11):e0277407. doi: 10.1371/journal.pone.0277407 (PMC9651549; doi:10.1371/journal.pone.0277407)
Supplement: S2 File — (PDF) [file pone.0277407.s003.pdf]

NIH. NATIONAL CANCER INSTITUTE – Center for Cancer Research

Tom Misteli, Ph.D. | Director

September 12, 2022

To whom it may concern:

This is to confirm that the clinical trial "Functional Imaging of Liver Cancer (FLIC): Study Protocol of a Phase 2 Trial of 18F-DCFPyL PET/CT Imaging for Patients with Hepatocellular Carcinoma" NCT05009979 under project ZIA BC 011800 has been funded in total by the NCI's intramural program through the Center for Cancer Research.

Tom Misteli, Ph.D.
CCR Director
